# DNA methylation outliers in normal breast tissue identify field defects that are enriched in cancer

**DOI:** 10.1038/ncomms10478

**Published:** 2016-01-29

**Authors:** Andrew E Teschendorff, Yang Gao, Allison Jones, Matthias Ruebner, Matthias W. Beckmann, David L. Wachter, Peter A. Fasching, Martin Widschwendter

**Affiliations:** 1 Department of Women's Cancer, University College London, Medical School Building, Room 340, 74 Huntley Street, London WC1E 6AU, UK; 2 CAS Key Lab of Computational Biology, CAS-MPG Partner Institute for Computational Biology, Shanghai Institute for Biological Sciences, Chinese Academy of Sciences, Shanghai 200031, China; 3 Statistical Cancer Genomics, Paul O'Gorman Building, UCL Cancer Institute, University College London, 72 Huntley Street, London WC1E 6BT, UK; 4 Department of Gynaecology and Obstetrics, University Clinic Erlangen, Friedrich-Alexander University Erlangen-Nuremberg, Erlangen 91054, Germany; 5 Department of Surgical Pathology, Institute of Pathology, University Clinic Erlangen, Friedrich-Alexander-University Erlangen-Nuremberg, Erlangen 91054, Germany

## Abstract

Identifying molecular alterations in normal tissue adjacent to cancer is important for understanding cancer aetiology and designing preventive measures. Here we analyse the DNA methylome of 569 breast tissue samples, including 50 from cancer-free women and 84 from matched normal cancer pairs. We use statistical algorithms for dissecting intra- and inter-sample cellular heterogeneity and demonstrate that normal tissue adjacent to breast cancer is characterized by tens to thousands of epigenetic alterations. We show that their genomic distribution is non-random, being strongly enriched for binding sites of transcription factors specifying chromatin architecture. We validate the field defects in an independent cohort and demonstrate that over 30% of the alterations exhibit increased enrichment within matched cancer samples. Breast cancers highly enriched for epigenetic field defects, exhibit adverse clinical outcome. Our data support a model where clonal epigenetic reprogramming towards reduced differentiation in normal tissue is an important step in breast carcinogenesis.

## Abstract (web summary)

Altered epigenetics is a feature of cancer but whether these changes occur early in tumour development is unclear. Here, the authors analyse methylation events in breast cancer and adjacent normal pairs, and show that methylation changes in the normal tissue are also found in the tumour, suggesting that some of these events occur early in cancer.

## Graphical abstract

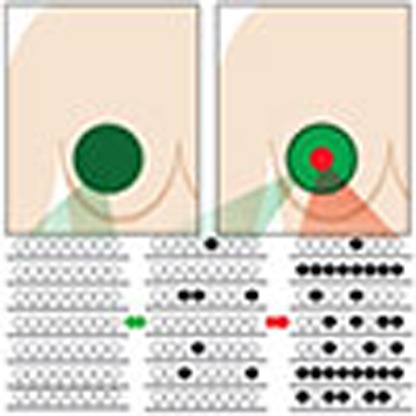

The realization that tumours are heterogeneous entities^1^,^2^,^3^, undergoing rapid evolution in response to intrinsic and extrinsic selection^4^,^5^,^6^,^7^, has given renewed importance to the study of cancer aetiology. Detecting molecular alterations, which happen early in carcinogenesis, is not only important for our understanding of carcinogenesis but also for implementing potential cancer prevention and early detection strategies. As access to the normal cells at the exact locations where cancers arise is naturally impossible, the only approach in humans to identify early molecular alterations is by comparison of normal tissue from healthy individuals to the normal tissue that is found adjacent to tumours. Such alterations, commonly known as field defects/effects^8^, have been predicted to exist mathematically^9^ and are thought to constitute the earliest clones in the carcinogenic process^10^. Previous experimental studies have described field defects in several cancers, including colorectal, prostate, breast, lung, oesophagus, stomach and skin^11^. More recently, a sequencing study identified genetic field defects in normal prostate tissue, with the specific mutations, also seen in the adjacent prostate cancers^12^. Although epigenetic field defects, notably DNA methylation alterations, have also been described^13^,^14^, it is still unknown how widespread epigenetic field effects are and how they contribute to carcinogenesis^11^.

Here we make significant progress towards answering these outstanding questions. By measuring DNA methylation in almost half a million CpG sites, and using a statistical paradigm for feature selection, we demonstrate the existence of widespread epigenetic field defects in normal tissues adjacent to breast cancers, with these defects becoming enriched in the progression to breast cancer. These data are consistent with a model in which epigenetic alterations predate the emergence of cancer.

## Results

### DNA methylation outliers identify field defects in breast cancer

**Figure 1:**
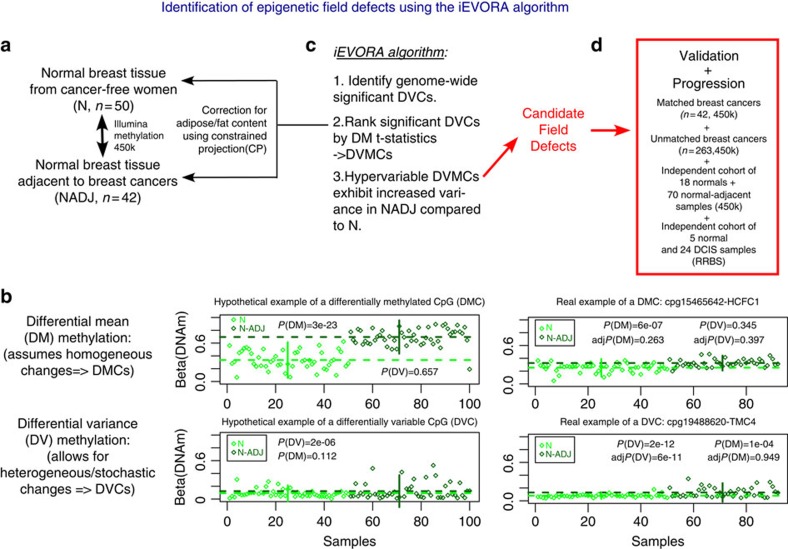
Overall analytic strategy for identifying and validating field defects in breast cancer. (**a**) Aim is to identify DNA methylation changes between normal tissue from cancer-free women (N) and age-matched normal samples adjacent to breast cancers (NADJ). This is done correcting for variable adipose/fat content across breast tissue samples and then performing a supervised analysis. (**b**) Two feature selection paradigms are possible: (i) a standard paradigm by which we select differentially methylated CpGs (DMCs) and which assumes homogeneity within the phenotypes being compared, (ii) a novel paradigm, based on the notion of differential variability (DV), which allows for heterogeneous/stochastic changes, and which identifies differentially variable CpGs (DVCs). Profiles to the left depict theoretical examples of a DMC and DVC. *P*-values are from a *t*-test for the case of assessing differential methylation (DM), and from a Bartlett's test for the case of assessing DV. Horizontal dashed lines indicate the mean DNA methylation value, vertical lines indicate the variance (±1.96 standard deviation). To the right, we depict real examples of a top ranked DMC and DVC derived from comparing N to NADJ samples. We give the *P*-values (*P*) and adjusted *P*-values (adj*P*: adjusted for multiple testing) for the case of *t*-tests (DM) and Bartlett's test (DV). Horizontal dashed lines indicate the mean DNA methylation value, vertical lines indicate the variance (±1.96 s.d.). Note that for the DVC, the main distinguishing feature is the variance, not the mean. (**c**) The iEVORA algorithm posits that relevant field defects are identified by first using a test for DV to select significant DVCs, and then ranking significant DVCs by a DM *t*-statistic. This results in differentially variable and differentially methylated CpGs (DVMCs). Those exhibiting increased variance in the NADJ samples represent candidate field defects. (**d**) Validation of field defects using matched and unmatched breast cancer samples, to assess if field defects progress or become enriched in the invasive cancer state, as well as validation of field defects in independent cohorts.

We generated Illumina 450k DNAm data in a large discovery set of breast tissue samples, including 50 normal/benign samples from cancer-free women, 42 matched normal–breast tumour pairs and an additional 263 breast cancers (Methods, Supplementary Tables 1 and 2). The data were of high quality, as assessed using a stringent quality control pipeline, with the top component of variation correlating with normal–tumour status (Methods, Supplementary Fig. 1). To identify epigenetic field defects, we initially restricted analysis to the 50 normal samples from cancer-free women and the 42 normal samples from age-matched female breast cancer patients (Fig. 1a). As adipose cells make a substantial component of normal breast tissue^15^, we first devised a statistical deconvolution algorithm to obtain sample-specific estimates of a sample's fat content (Methods). The algorithm was successfully validated on independent adipose tissue data (Methods, Supplementary Table 3). Singular value decomposition over the 92 normal samples confirmed that the top component of variation strongly correlated with a sample's fat content (Supplementary Fig. 2). However, fat content did not vary significantly between the normal and normal-adjacent samples (Supplementary Fig. 3A), and adjustment for fat content did not have a major impact on differential methylation analysis, with differentially methylated CpGs between normal and normal-adjacent tissue not attaining genome-wide significance (minimum false discovery rate (FDR)=0.3) in either adjusted or unadjusted analysis (Supplementary Fig. 3B).

**Figure 2:**
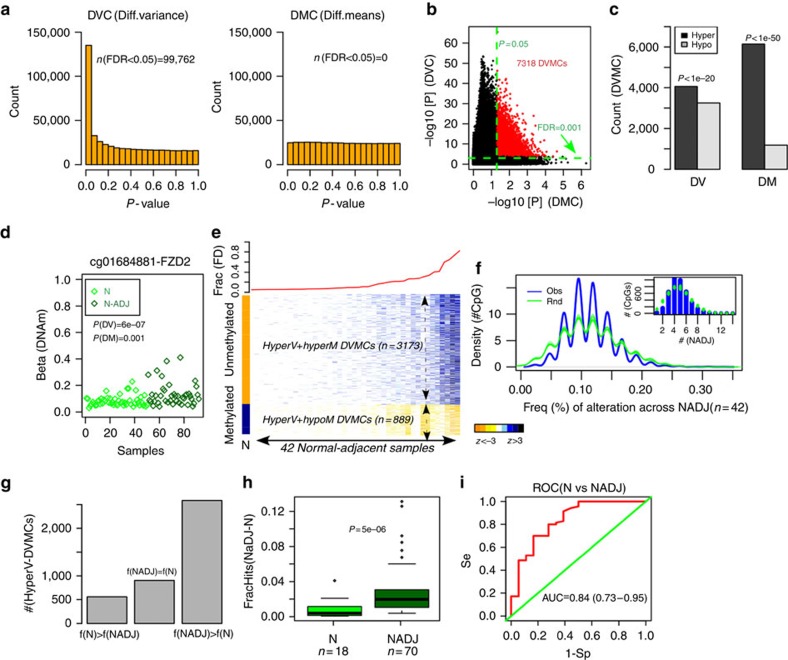
Identification of stochastic DNA methylation field defects in breast cancer and validation. (**a**) Histograms of *P*-values from Bartlett's test (DVC-differentially variable CpGs) and *t*-tests (DMC-differentially methylated CpGs) comparing 50 normal breast tissue samples from healthy women to 42 normal-adjacent samples from breast cancer patients. The number of probes passing an FDR-corrected threshold of 0.05 are shown. (**b**) Definition of the 7,318 differentially variable and differentially methylated CpGs (DVMCs), as those probes passing an FDR threshold for DV of 0.001 and an uncorrected *P*-value threshold for DM of 0.05. (**c**) Relative numbers of DVMCs that are hyper-or-hypovariable (DV), and hyper-or-hypomethyled (DM). Binomial *P*-values are given. (**d**) The DNAm profile of a hypervariable+hypermethylated DVMC. *Y* axis labels the DNA methylation beta-value, *x* -axis labels the samples. *P*-values shown are for a Bartlett's test, which tests for DV, and for a *t*-test, which tests for differential average methylation (DM). (**e**) Upper panel: fraction of hypervariable DVMCs significantly altered in each normal-adjacent sample, with samples ordered in increasing order. Left colour bar depicts the average DNAm beta-value of the hypervariable DVMCs across the 50 normal samples from cancer-free women. Orange: beta-value<0.2, blue: beta-value>0.6. Heat map depicts the *z*-scores of differential DNAm change for each DVMC and normal-adjacent sample relative to the normal state, with samples ordered according to the overall fraction of alteration. (**f**) Density-histogram plot of the number of hypervariable DVMCs exhibiting a given fraction of DNAm alterations across the 42 normal-adjacent samples (blue curve). In green, we show the density obtained from Monte-Carlo randomization. Inlet figure depicts the same data, but using absolute numbers of CpGs (*y* axis) and actual numbers of normal-adjacent samples. (**g**) Relative numbers of hypervariable DVMCs shown in heat map of **e**, which show a lower [f(N)>f(NADJ)], equal [f(NADJ)=f(N)] or higher frequency of alteration [f(NADJ)>f(N)] in an independent set of normal adjacent (NADJ) samples compared with normals (N). (**h**) Box plot comparing the frequency of alteration of hyper-DVMCs (*y*-axis: FracHits) in the independent set of normal-adjacent (NADJ) and normal (N) samples. *P*-value is from a Wilcoxon rank sum test. (**i**) Receiver operating curve (ROC)-curve and AUC value plus 95% confidence interval corresponding to **h**.

Owing to the evidence that DNA methylation changes in normal cells at risk of undergoing morphological transformation exhibit largely stochastic and heterogeneous patterns^16^, we posited that identification of DNAm field defects would benefit from a feature selection algorithm that recognizes such heterogeneity, for instance one based on differential variability (DV, Fig. 1b)^16^,^17^. We developed a novel DV algorithm, called iEVORA, (Methods, Fig. 1c), which identified a total of 7,318 differentially variable and differentially methylated CpGs (DVMCs; that is, ∼1.5% of the interrogated sites, FDR< 0.001, Supplementary Data 1 and Fig. 2a,b), with the majority of these DVMCs being more variable (4,062/7,318, ∼56%) and hypermethylated (6,138/7,318, 84%) in the normal samples adjacent to breast cancers (Binomial test *P*-values: *P*<1e-20, Fig. 2c). A typical DNAm profile of a hypervariable and hypermethylated DVMC, illustrates how increased variance, and not increased mean DNAm level, is the main distinguishing characteristic (Fig. 2d). Indeed, increased variance in the normal-adjacent state was observed to be driven by a relatively small number of outlier samples exhibiting ‘jumps' in DNAm beta-values of the order of 20–30% (Fig. 2d). We were able to confirm this for the great majority of hypervariable DVMCs, with the outlier samples exhibiting hyper or hypomethylation, depending on the average beta-value in the normal samples from cancer-free women (Supplementary Fig. 4). Further attesting to the importance of considering DV, DVMCs were also highly robust to correction for variable adipose cell content (Supplementary Fig. 5), indicating that these DVMCs mark DNAm changes in the epithelial or stromal cell compartment of the normal-adjacent samples. Analysing the distribution of DVMCs according to specific gene regions, we observed that among the hypermethylated DVMCs, hypervariable ones were much more likely to map to regions within 1.5 kb upstream of the transcription start site (TSS), whereas hypovariable ones were more likely to map to the gene body (Fisher-test *P*-value: *P*<1e-15, Supplementary Table 4), consistent with DNAm levels in healthy normal cells starting out low near the TSS and high in gene bodies. Among the hypomethylated DVMCs, hypervariable ones were overrepresented in the gene body, whereas hypovariable ones mapped preferentially to the 5′untranslated region (Fisher-test *P*-value: *P*<1e-15, Supplementary Table 4), again consistent with the baseline/normal levels of DNAm in these regions being high and low, respectively.

We observed that the 42 normal adjacent samples exhibited marked differences in the overall number of epigenetic alterations at hypervariable DVMC loci, with only 2 samples exhibiting alterations at more than 50% (that is, 2,031) of the loci (Fig. 2e). The great majority of samples exhibited alterations at less than 5% of these loci (that is,<200). From the perspective of individual DVMC loci, the overall frequency of alteration across the 42 samples was also low, with the most frequently altered CpG attaining significant hypermethylation in only 14/42 (33%) of samples (Fig. 2f). The overwhelming majority of DVMCs showed DNAm changes in only four to five normal-adjacent samples, supporting the view that DNAm changes in the normal-adjacent tissue are largely stochastic when assessed across unrelated individuals (Fig. 2f). We confirmed, by Monte-Carlo analysis, that the observed number of CpGs exhibiting the largest frequencies of alteration were not higher than that expected by random chance (Fig. 2f).

Although from the perspective of any one of the hypervariable DVMCs, the field defects appear largely stochastic across different women, it could be that normal-adjacent samples carrying a higher fraction of field defects are associated with specific tumour characteristics. To study this, we correlated the fraction of epigenetic alterations at hypervariable DVMC loci in each normal-adjacent sample to a range of clinical characteristics of the matched tumours, including estrogen receptor (ER) progesterone receptor (PR) human epidermal growth factor receptor 2 (HER2) status, as well as age, tumour size, KI67, stage, grade, nodal and menopausal status. A strong association was only observed for stage, with stage-2 tumours exhibiting a significantly higher load of epigenetic changes at the hypervariable DVMC loci compared with stage-1 cancers (Supplementary Fig. 6).

### Validation of epigenetic field defects in an independent cohort

To validate the identified field defects, we collected an independent cohort of normal breast samples (*n*=18, obtained from reduction mammoplasty) and normal samples adjacent to invasive breast cancers (*n*=70), for which Illumina Infinium 450k DNAm data were available (Fig. 1d)^18^. We observed that the top component of variation in this data set exhibited an almost perfect correlation with our *in-silico* adipose cell content estimate (Supplementary Fig. 7). For each sample and hypervariable DVMC, we estimated whether it exhibited a significant deviation in DNAm relative to the 18 normal samples, adjusting for potential variation in adipose cell fractions. Of the 4,062 hypervariable DVMCs (3,173 hypermethylated+889 hypomethylated) from the discovery set, 4,047 (that is, over 99%) were present in this independent study, with 2,586 (64%) exhibiting higher frequencies of alteration in normal-adjacent samples compared with normal samples, with 22% not showing any difference and with 14% exhibiting fewer alterations in normal-adjacent samples, thus demonstrating that a substantial fraction of the identified field defects are reproducible (Fig. 2g). The normal-adjacent samples exhibited fractions of alteration at the hypervariable DVMC loci, similar to the fractions in the discovery set, and significantly higher than those of the normal samples (Fig. 2h), allowing normal-adjacent from normal samples to be discriminated with an area under the curve (AUC) of 0.84 (Fig. 2i).

### Field defects exhibit progressive changes in breast cancer

**Figure 3:**
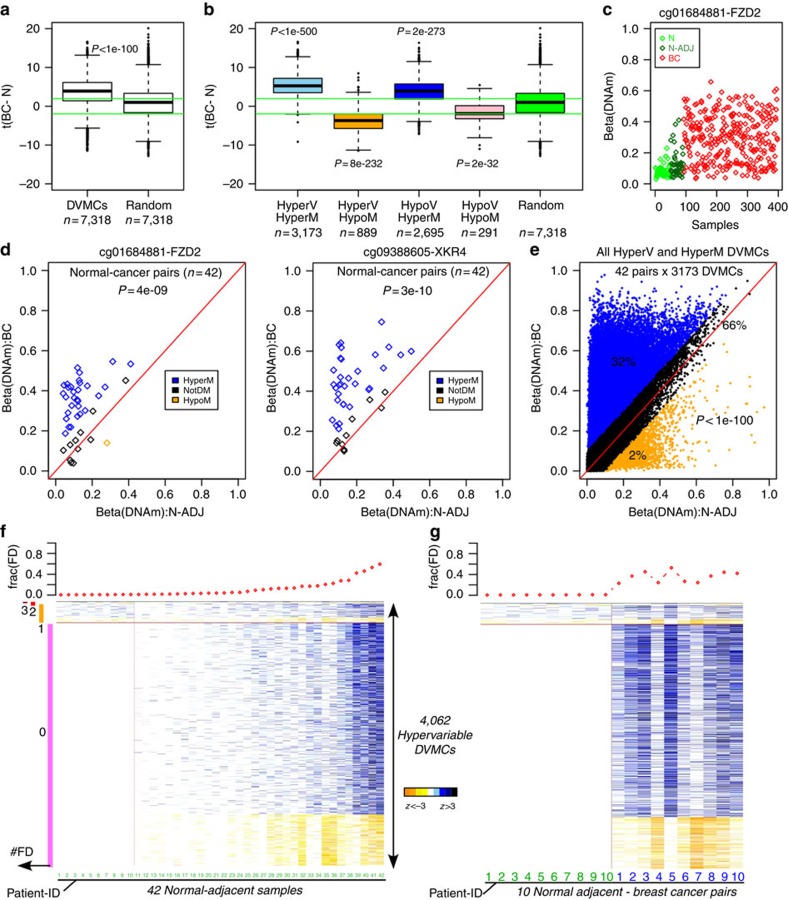
Progression of field defects in breast cancer. (**a**) Box plot of *t*-statistics of differential DNA methylation between the 305 breast cancer (BC) patients and the 50 normals (N) from healthy women (*y* axis: t(BC-N)) for the 7,318 DVMCs, compared with a random selection of CpGs. Positive *t*-statistics indicate larger DNAm values in BC compared with N. Wilcoxon-rank sum test *P*-value is given. Horizontal green lines indicate the lines of *P*=0.05. (**b**) As **a**, but with the DVMCs broken up into four categories, according to whether CpG is hypervariable or hypovariable and whether the difference in mean DNA methylation is increased (hypermethylated) or decreased (hypomethylated) in the 42 normal adjacent samples compared with the 50 true normals. (**c**) Example of a DNAm profile of a hypervariable and hypermethylated DVMC, showing the progressive change in DNA methylation. (**d**) Scatter plots of DNA methylation for two DVMCs, restricting to the 42 matched normal–tumour pairs, with *x* axis labelling the beta-value in the normal adjacent sample, and *y* axis labelling the corresponding beta-value in the matched breast tumour. Paired Wilcoxon test *P*-values are given. Blue and orange points represent breast cancer patients for which the change in mean DNA methylation between normal-adjacent and cancer was larger than 0.1 in absolute terms, with blue indicating hypermethylation and orange hypomethylation. (**e**) As **d**, but now for all 3,173 hypervariable and hypermethylated DVMCs superimposed on same plot. We provide the proportion of data points that exhibit hypermethylation (blue), no significant changes (black), and hypomethylation (orange). (**f**) Top plot shows the fraction of hypervariable DVMCs (aka field defects, frac(FD)) significantly altered in each normal-adjacent sample, with samples ordered in increasing order. Left colour bars mark the DVMCs with the number of significant DNAm alterations that they exhibit across the ten samples exhibiting the lowest fractions of alterations. Heat map depicts the *z*-scores of differential DNAm change for each DVMC and normal-adjacent sample relative to the normal-state, with samples ordered according to the overall fraction of alteration. (**g**) As **f**, but now restricting to the ten normal-adjacent breast cancer pairs corresponding to the ten patients with the lowest fractions of DNAm alterations in normal-adjacent tissue.

If the DNAm defects identified in the normal adjacent tissue mark cells that are important for the development of breast cancer, we would expect to see that these loci exhibit larger differences in DNAm when comparing breast cancers (presumably enriched for these cells) to the normal samples of healthy subjects. To test this, we computed *t*-statistics of differential methylation between the 305 breast cancers and the 50 normal samples from healthy subjects, for all previously identified DVMCs. We observed that the great majority of DVMCs exhibited significant differential DNAm changes in breast cancer and that they exhibited much stronger associations than a randomly selected set of CpGs (Fig. 3a), clearly supporting the view that the identified DVMCs are marking cells that progress to breast cancer. Among all DVMCs, those hypervariable in normal adjacent samples exhibited the correspondingly strongest changes in DNA methylation in breast cancer (Fig. 3b), attesting to their special importance in cancer development. A plot of the DNAm profile of a representative DVMC in this class confirmed a pattern of progressive DNAm change, with a much higher proportion of breast cancers exhibiting increased deviations in DNAm from the normal state (Fig. 3c).

To explore the patterns of progression in more detail, we compared DNA methylation levels of representative DVMCs within each of the 42 matched normal–tumour pairs. DVMCs hypermethylated in normal adjacent tissue also exhibited statistically significant increases in DNA methylation in the matched breast cancers (Wilcoxon rank sum Paired test: *P*<1e-8, Fig. 3d). Confirming the generality of this, we observed that up to 32% of the hypervariable and hypermethylated DVMCs underwent further significant increases in DNA methylation in breast cancer, in contrast to only 2% exhibiting a reversal in DNAm change (that is, hypomethylation; Fisher's exact test, *P*<1e-100, Fig. 3e). Thus, a substantial proportion of the DVMCs hypermethylated in normal-adjacent tissue relative to the normal samples of cancer-free women, continue to exhibit further DNAm increases in the matched breast cancers. In contrast, the 2% of DVMCs exhibiting hypomethylation likely represent either subclones whose relative representation in the breast cancer is reduced or sites undergoing active DNA demethylation in cancer. We note that none of the other three categories of DVMCs exhibited as consistent patterns of progression in the breast cancers as those of hypervariable and hypermethylated DVMCs (Supplementary Fig. 8).

### Convergence of DNA methylation defects in breast cancer

Interestingly, although the identification of hypervariable DVMCs was driven by a relatively small number of normal-adjacent samples exhibiting widespread deviations at these loci (Figs. 2e and 3f), most matched breast cancer samples exhibited increased DNAm deviations at the same CpGs. To explore this further, we identified the ten normal-adjacent samples that exhibited least DNAm changes at the identified hypervariable DVMCs (Fig. 3f). The overwhelming majority of the DVMCs did not exhibit any significant deviations in DNAm across these ten samples (comparing to the 50 normal healthy ones), with a relatively few loci exhibiting 1, 2 and 3 changes (Fig. 3f). Remarkably, however, the ten breast cancers matched to these normal-adjacent samples did exhibit significant DNAm deviations at a large proportion of these same loci (Fig. 3g), indicating that although these particular DNAm alterations were not strongly represented in the normal-adjacent tissue, that they did become enriched in the actual cancers. This suggests that although the DNAm landscape of normal-adjacent tissue is highly heterogeneous across different patients, that the corresponding landscape in the paired breast cancers is more homogeneous in the sense that any two breast cancers will share a larger fraction of loci exhibiting significant DNAm deviations from the normal state. Thus, although any two randomly selected normal-adjacent samples differ substantially in terms of the specific loci exhibiting significant DNAm deviations, any two randomly selected breast cancers will share a higher fraction of such loci (Supplementary Fig. 9A), consistent with our previous observations in cervical cancer^19^. To formally test this again here, we computed the Manhattan distance of significant DNAm deviations over the hypervariable DVMCs for each pair of normal-adjacent samples, and then again for each pair of matched breast cancers, comparing the resulting distances against their corresponding null distributions obtained by randomizing the deviation matrices (keeping the frequencies of alteration per sample fixed, Methods). This confirmed that pairs of breast cancers showed smaller Manhattan distances than random, indicating a higher than random overlap of DVMCs undergoing significant DNAm deviations, whereas the Manhattan distance for normal-adjacent samples was more consistent with that of a random distribution (Supplementary Fig. 9B).

### Field defects are present in breast ductal carcinomas *in situ*

We would expect the identified field defects to also be present in lesions that often precede invasive breast cancer. To test this, we first obtained Illumina Infinium 450k data for an independent cohort of 15 normal-adjacent and 40 ductal carcinomas *in situ* (DCIS) of the breast^20^. Mapping our hypervariable and hypermethylated DVMCs in this data, we observed that these sites exhibited substantial DNA methylation increases in DCIS compared with normal-adjacent samples (Wilcoxon rank sum test: *P*<1e-10, Supplementary Fig. 10). To further test this, we obtained another independent set of 5 normal breast samples (from reduction mammoplasias) and 24 high-grade pure DCIS samples, for which reduced-representation bisulfite-sequencing (RRBS) data were available^21^. Of the 3,173 hypervariable and hypermethylated DVMCs, 983 had RRBS data (20-fold coverage) within a 100-bp window on either side of the DVMC. Computing the average deviation in DNA methylation for the DCIS samples relative to the 5 normal samples over these 983 sites, we could confirm that these DVMCs exhibited increased DNAm levels in the DCIS (Wilcoxon rank sum test: *P*<1e-4, Supplementary Fig. 11).

### Field defects are enriched for gene regulatory elements

**Figure 4:**
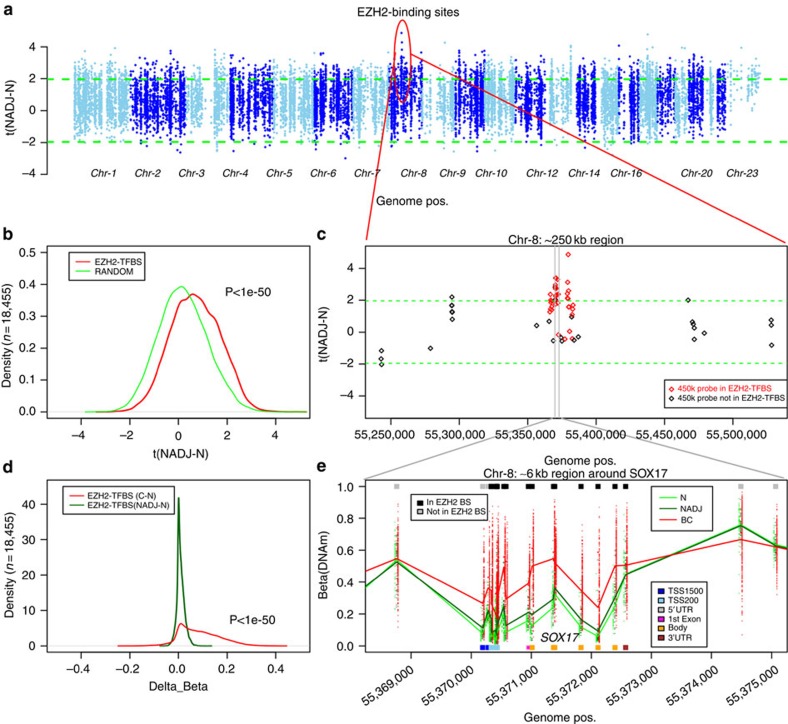
Enrichment of EZH2 TF-binding sites among DNA methylation field defects. (**a**) Manhattan type plot of the *t*-statistics of differential DNA methylation between normal adjacent (NADJ) and normal breast tissue (N; *y* axis: t(NADJ-N)) of 450k probes mapping to EZH2-binding sites (18,455 sites). Green dashed lines represent the lines corresponding to *P*=0.05. Positive values indicate larger DNAm in NADJ tissue compared with normals. (**b**) Density distribution of the *t*-statistics of differential methylation between normal adjacent (NADJ) and normal breast tissue (N) of 450k probes mapping to EZH2-binding sites (red) compared with a randomly chosen set (green). *P*-value is from a Wilcoxon rank sum test. (**c**) Zoomed in version of **a** focusing on a 250-kb region on chromosome-8, but now showing all 450k probes in the region, with those mapping to EZH2-binding sites indicated in red. (**d**) Comparison of the density distribution of average differences in DNA methylation (*x* axis: delta_Beta) for the 18,455 probes mapping to EZH2-binding sites (EZH2 TFBS) between cancer and normal adjacent tissue (C-N), to the corresponding differences between normal adjacent and normal tissue (NADJ-N). Thus, positive delta_Beta values correspond to higher DNAm values in cancer compared with normal (C-N), or to higher DNAm values in NADJ tissue compared with normal (NADJ-N). (**e**) DNA methylation beta values for all samples and probes in an ∼6-kb region, centred around the PRC2 target, *SOX17*. The lines represent the mean DNAm values in each group: normal (N), normal-adjacent (NADJ) and breast cancer (BC). Probes/CpGs have been annotated according to whether they fall in a EZH2-binding site, and which gene region they map to.

Having established the existence of widespread DNAm field defects in breast cancer, we next explored their distribution in relation to regulatory elements, including transcription factor-binding sites^22^,^23^. Using ENCODE/NIH Roadmap ChIP-Seq data for binding sites in human embryonic stem cells^22^,^24^,^25^, we assessed enrichment of 58 transcription factors among the four categories of DVMCs. We only found enrichment among the hypervariable and hypermethylated DVMCs (Supplementary Data 2), with the top five transcription factors including two Polycomb Repressive Complex 2 (PRC2) members (EZH2 and SUZ12) as well as RBBP5, CTCF and RAD21. This is similar to the enrichments we found previously in relation to smoking^26^ and ageing^23^. Plotting the statistics of differential methylation between normal-adjacent and normal tissue for all 450k probes mapping to EZH2- and CTCF-binding sites, confirmed a statistically significant trend for these sites to become hypermethylated in normal-adjacent tissue (Fig. 4a–c and Supplementary Fig. 12A–C). Importantly, EZH2-binding sites also exhibited progressive increases in DNAm in breast cancer compared with normal-adjacent tissue (Fig. 4d), and although this was also true for CTCF-binding sites, the changes were more subtle (Supplementary Fig. 12D). Examples of a PRC2 target, *SOX17*, and of a DNase Hypersensitive region containing a CTCF-binding site upstream of histone HIST1H4D illustrate the progressive DNAm increases from normal to normal adjacent tissue to breast cancer (Fig. 4e and Supplementary Fig. 12E). Gene set enrichment analysis (GSEA) of DVMCs mapping to either the TSS1500, TSS200 or the first exon regions (regions containing 450k probes which are most informative of gene expression^27^), confirmed that the strongest enrichment of any biological term was for hypervariable and hypermethylated DVMCs, which were strongly enriched for bivalently or PRC2 marked genes (Hypergeometric test: *P*<1e-40, Supplementary Data 3 and Supplementary Fig. 13).

### Coordinated field defects target the wingless related iNTegration (WNT)/fibroblast growth factor (FGF) signalling

**Figure 5:**
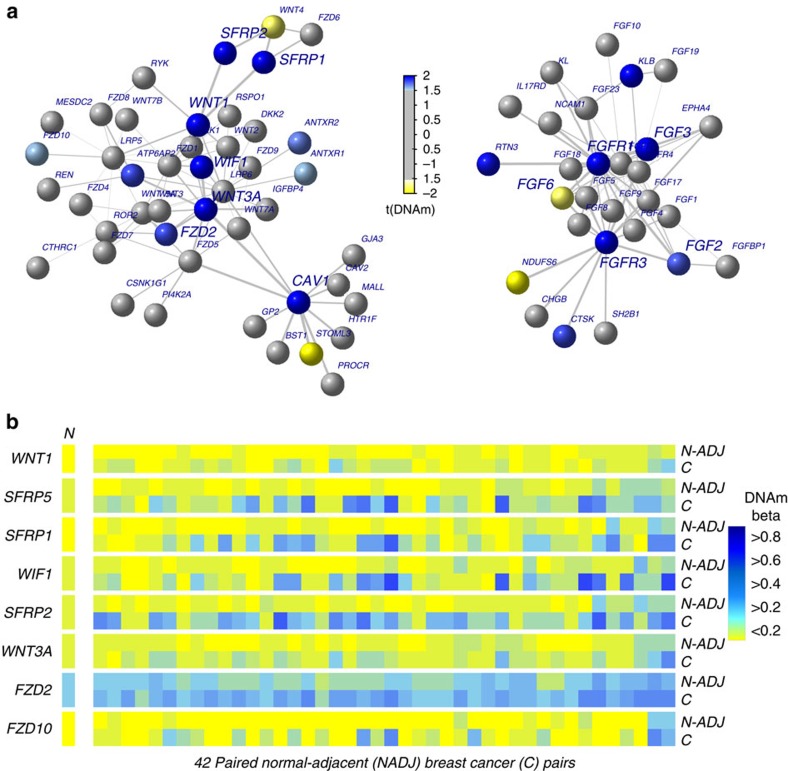
Field defects are enriched for WNT and FGF signalling pathways. (**a**) Examples of two interactome hotspots of epigenetic deregulation comparing normal-adjacent to normal-healthy samples, inferred using the EpiMod/FEM algorithm. In colour, we indicate the nodes exhibiting significant DNA methylation changes. (**b**) Heat maps of DNA methylation of WNT-signalling pathway members for the 42 matched normal-adjacent (N-ADJ) breast cancer (BC) pairs. In the case of the normal samples from healthy subjects (N), we show the average DNA methylation values across all 50 samples.

Next, we asked if the identified epigenetic field defects also target specific gene modules or signalling pathways. To address this we used a functional supervised algorithm, called FEM (Functional Epigenetic Modules)^27^,^28^,^29^, which seeks hotspots of differential promoter DNA methylation in the context of a human interactome. This revealed a number of significant hotspots of epigenetic deregulation, centred around important breast cancer genes, including *FOXA2, PAX6* and *L1CAM* (Supplementary Data 4), with the two largest modules mapping to the WNT and FGF signalling pathways, respectively (Fig. 5a and Supplementary Data 4). The promoters of many pathway members were hypermethylated in normal-adjacent tissue relative to normal tissue from cancer-free women, with most of these gene promoters undergoing further progressive increases in DNA methylation in the matched 42 breast cancers (Fig. 5b and Supplementary Fig. 14). Within a signalling pathway, alterations in one pathway member in one normal adjacent sample were usually accompanied by changes in other pathway members within the same sample, indicating coordinated changes (Fig. 5b and Supplementary Fig. 13). We confirmed, statistically, that epigenetic changes in the normal adjacent tissue and within the signalling pathway, generally, exhibited a level of coordination (Supplementary Fig. 15 and Methods).

### Enrichment level of field defects correlates with phenotype

**Figure 6:**
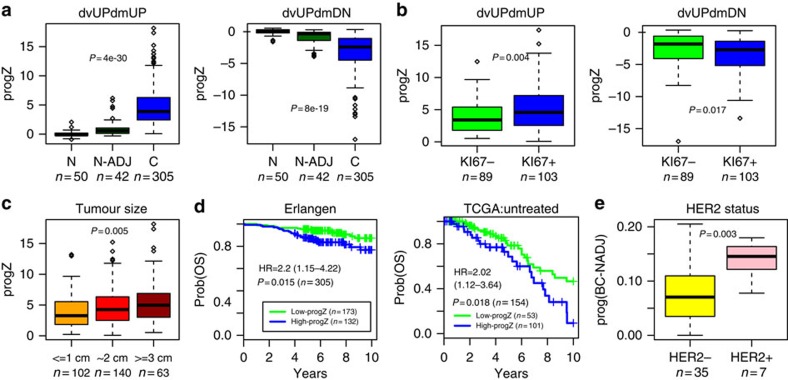
Progression of field defects correlate with proliferation and overall survival. (**a**) Box plots of progression *Z*-scores against sample status (N=normal-healthy, NADJ=normal adjacent, C=breast cancer) for each class of DVMC. *P*-values are from a linear regression. (**b**) Box plots of the same progression Z-scores against the proliferation index (KI67) for each class of DVMC. *P*-values are from a Wilcoxon-rank sum test. (**c**) Box plot of the progression *Z*-score of the DVMCs hypervariable and hypermethylated in normal-adjacent compared with normal-healthy, against tumour size. *P*-value is from a linear regression. (**d**) Kaplan–Meier survival curves for breast tumours stratified into groups of low and high progression *Z*-scores. Hazard ratio, 95% confidence interval and Cox-regression *χ*^2^ test *P*-value are given. Groups were obtained by clustering the progression *Z*-scores of all samples into two groups using the pam-algorithm from the *cluster* R package. (**e**) Box plot of the individualized progression deviation score for each of the 42 breast cancer patients with a matched normal-adjacent sample, against HER2 status. *P*-value is from a Wilcoxon rank sum test.

We reasoned that breast tumours highly enriched for cells marked by epigenetic field defects would correspond to tumours with a higher clonogenic potential, exhibit increased proliferation rates and adverse outcome. To test this, we first estimated for each breast cancer sample an enrichment or ‘progression' Z-score, which measures the average deviation in DNA methylation at DVMC loci from the 50 normal-healthy samples (Methods). This analysis was performed separately for the four categories of DVMC loci considered earlier. First, we verified that these progression scores were higher in cancer compared with normal-adjacent tissue (Fig. 6a and Supplementary Fig. 16A). We note that the scores exhibited stronger trends for the hypervariable DVMCs supporting the biological significance of the hypervariability seen in the normal-adjacent samples (Supplementary Fig. 16A). In correlating the progression Z-scores of the breast cancers to clinical tumour features, including ER, PR and HER2 status, as well as KI67, tumour size, age and clinical outcome, we observed a significant positive association with proliferation (KI67; Fig. 6b, Supplementary Fig. 16B and Supplementary Table 5), which was strongest for the hypervariable and hypermethylated DVMCs. Interestingly, the score computed at these specific sites also correlated significantly with tumour size (Fig. 6c and Supplementary Table 5) and clinical outcome (Fig. 6d and Supplementary Table 5). The association with survival remained marginally significant in a multivariate model, which included age, stage, ER-status and tumour size (Supplementary Table 6). Similar results were observed had we used a progression ‘hit' score estimated from counting the fraction of hypervariable DVMC loci exhibiting significant deviations in DNAm (Supplementary Table 7). Interestingly, we observed that the association with outcome was stronger when we restricted to the untreated subset, although significance was not reached due to small sample size (Supplementary Fig. 17). We therefore tried to validate the association of this progression score in an independent set of untreated breast cancer patients from TCGA (The Cancer Genome Atlas)^30^. Importantly, the only progression score to validate in the TCGA was the one based on the hypervariable and hypermethylated DVMCs, consistent with the results in our cohort (Fig. 6d and Supplementary Table 8). Finally, we also computed a personalized progression score, specifically, for each of the 42 breast cancers relative to their matched normal-adjacent tissue. Interestingly, for the hypervariable+hypermethylated DVMC class, we observed a significant association with HER2 status, indicating that breast cancers which exhibit stronger progressive DNAm deviations from their normal-adjacent tissue are more likely to be HER2+ breast cancers (Fig. 6e and Supplementary Table 9).

## Discussion

Previous to this work, the frequency of epigenetic field defects in breast cancer was largely unknown. Had we adopted a statistical feature selection framework based on the common paradigm of differential methylation, our genome-wide analysis would have concluded, erroneously so, that there are no genome-wide significant DNA methylation field defects in breast cancer. However, motivated by our previous work in cervical cancer, which points to a fundamentally stochastic nature underlying the earliest of epigenetic changes^16^,^17^, we here hypothesized that a feature selection paradigm based on DV would improve the sensitivity to detect heterogeneous, stochastic, DNAm changes. We further posited that these DNAm changes would mark cells in the normal-adjacent tissue, which drive carcinogenesis. Indeed, we have here shown how our novel statistical algorithm identified thousands of genomic sites, which exhibited increased variability in DNAm within the normal-adjacent samples, compared with normal breast tissue from age-matched cancer-free women. In addition to validating these field defects in an independent cohort, we have also demonstrated that the hypervariable DVMCs exhibit progressive DNAm changes in the breast cancers of the same women who provided a normal sample adjacent to their tumours, clearly supporting the view that the identified epigenetic field defects are marking early cancer precursor cells, which become enriched in the invasive cancers.

Importantly, we have also shown that the DNAm changes marked by the hypervariable DVMCs are not the result of variations in adipose cell content, a potential major confounder in these analyses. Although we cannot exclude for sure that some of the DV may be driven by changes in the stromal compartment, including variations in immune cells, this is highly unlikely for the following reasons. First, the DNAm changes driving the DV between the normal samples from cancer-free women and the normal-adjacent samples involve beta-value changes typically on the order of at least 20–30% (if not higher). As we have excluded variations in fat/adipose cell content as causing these changes in DV (despite the fact that changes in adipose cell content do carry most of the correlative variation in the data), it is therefore highly unlikely that changes in the stromal cell compartment could account for these rather big changes (>20–30%) in DNAm between the two normal phenotypes. Second, the GSEA on genomic pathways and TFBS did not reveal any strong enrichment of terms or TFs which are specific to immune cells or other stromal cells, suggesting that the DVMCs are not markers of these cell types. Thus, the DV we observe is most likely driven by changes in the epithelial cell compartment of the breast tissues.

The epigenetic field defects we have observed represent heterogeneous, that is, infrequent, events across the 42 normal-adjacent samples. This suggests that early epigenetic events in breast cancer are marked by a stochastic component, as otherwise, the observed frequency of alteration in normal-adjacent samples would have been much higher. On the other hand, we have also clearly demonstrated that epigenetic field defects do not happen randomly in the genome: binding sites of transcription factors important in specifying chromatin architecture, notably members of the PRC2 complex (EZH2, SUZ12), CTCF and RAD21, were all observed to acquire DNA methylation in normal cells adjacent to breast cancers, and to exhibit even further DNAm increases in the actual cancer. A system-level gene promoter analysis also confirmed that epigenetic field defects are not entirely random, targeting specific gene modules and signalling pathways, notably WNT signalling, a stem-cell differentiation pathway which has been observed to be epigenetically deregulated in other cancer types, including pre-neoplastic lesions^31^,^32^,^33^. The results of an ordinary GSEA also supported the role of WNT signalling, as well as FGF signalling, with one of the most highly ranked terms by odds ratio containing several genes previously implicated in mammary tumorigenesis and mapping to both of these pathways^34^ (Supplementary Table 4).

In summary, epigenetic field defects in breast cancer are widespread. Although specific defects are infrequent and appear largely stochastic across individuals, their genomic distribution is highly non-random affecting binding sites of transcription factors specifying chromatin architecture and stem-cell differentiation pathways. As with genetic mutations, alterations in DNA methylation also appear to mark pre-neoplastic normal cells that later transform and become enriched in cancer.

## Methods

### Primary DNA methylation dataset

A total of 397 breast tissue samples were collected within the Bavarian Breast Cancer Cases and Controls Study 2 (here denoted as the Erlangen data set). This study was approved by the Ethics Committee of the Medical Faculty, Friedrich-Alexander University (Ref.No.4514) and all patients gave written informed consent. The 397 samples included 50 normal/benign tissue specimens from 50 healthy women, 42 matched normal-breast cancer samples (normal samples being adjacent to the tumours, so a total of 84 samples) and 263 unmatched breast cancers. For sampling of adjacent normal and cancer tissue, a minimal distance from the cancer margin to the area of the breast from which the normal adjacent tissue was taken was 3 cm. Care was taken that the normal areas represented macroscopically healthy breast tissue. The samples (cancer and normal) were snap frozen and stored at −80 °C. For DNA extraction, sections (3 × 10 μm) of the fresh frozen tissues were done. Tumour tissues contained >70% cancer tissue and no cancer tissue was present in adjacent normal samples. DNA was extracted and processed following standard procedures for the Illumina 450k DNA methylation beadarrays^35^. Demographic and clinical details of the women and breast cancers are provided elsewhere (Supplementary Tables 1 and 2).

### Normalization of DNA methylation data

Raw Illumina data files were processed with the *minfi* Bioconductor package^36^. Using the *P*-values of detection from this package, we next estimated coverage of each sample and probe. All samples and probes had reasonable coverage. *P*-values larger than 0.05 were assigned not available (NAs) and later imputed using the *impute.knn* (k=5) function from the *impute* Bioconductor package^37^. To adjust for the well-known bias of type-2 probes, we ran each sample through BMIQ^38^. This completed the intra-sample normalization, resulting in a DNAm beta-valued data matrix of 485,512 probes and 397 samples. Inter-sample effects were assessed using a Singular Value Decomposition approach, as implemented by us previously, to evaluate the relative amounts of variation correlating with biological and technical factors^39^. Biological factors (that is, normal/tumour status, grade) were prominent among the top singular vectors with no major batch or chip effects causing potential confounding. Data are available on GEO (accession number GSE69914, *www.ncbi.nlm.nih.gov/geo*). We further note that although we did not remove the 93,382 cross-reactive/polymorphic 450k probes of Chen *et al*.^40^, that we did test the robustness of all results presented in this study against removal of such probes from our list of DVMCs (see below).

### Supervised analysis using the iEVORA algorithm

Owing to the more likely stochastic nature of epigenetic changes in early pre-neoplastic lesions^16^,^17^, we performed feature selection using a novel statistical algorithm based on the concept of DV. Specifically, we developed a regularized version of the feature selection procedure implemented in our previous EVORA (Epigenetic Variable Outliers for Risk prediction Algorithm) algorithm^16^, called iEVORA. Briefly, we subjected each CpG to a Bartlett's test to identify those whose DNA methylation patterns are differentially variable between the 50 normal samples and the 42 normal samples adjacent to tumours. Those CpGs that passed a stringent FDR<0.001 threshold, as estimated with the *q*-value Bioconductor package^41^, were designated differentially variable CpGs (DVCs). Because Bartlett's test is overly sensitive to single outliers, we regularized the procedure by re-ranking DVCs according to their differential DNA methylation *t*-statistic, and selecting those with a *t*-test unadjusted *P*-value of less than 0.05. We call this subset of DVCs, differentially variable and DVMCs. The top ranked DVMCs are differentially variable, but those with the larger *t*-statistics are ranked highest, a procedure that penalizes DVCs driven by only a few outliers. R-code implementing the iEVORA algorithm is freely available as an executable R-script *‘iEVORA.R'*, which is provided as Supplementary Software 1.

### DVMCs and cross-reactive/polymorphic probes

Chen *et al*. identified a total of 93,382 cross-reactive/polymorphic 450k probes, which could result in unreliable measurements. This constitutes about 19% of the total number of probes on the Illumina 450k beadarray. We verified that among our list of 7,318 DVMCs, there were only 923 overlapping with Chen's list. This is far less than the number expected to overlap by random chance (0.19 × 7,318=1,407), indicating that our DVMCs are underenriched for potentially problematic probes. This underenrichment was particularly strong for the hypervariable+hypermethylated DVMCs, as of the 3,173 of such DVMCs, only 208 were problematic, whereas by random chance we would have expected up to 610 to be cross-reactive or polymorphic. Thus, this confirms that DV is selecting biological features, in fact, avoiding the selection of potentially problematic probes. We further note that all analyses in this study were reproducible after removal of the 923 overlapping crossreactive/polymorphic probes from our DVMC list.

### Correction for intra-sample adipose cell content

As adipose/fat cells are known to be a major component of breast tissue, we devised a statistical algorithm to deconvolve the effects of contaminating fat cells. The algorithm uses Illumina 450k DNAm reference profiles for human mammary epithelial cells (HMECs) and adipose tissue, which we obtained from ENCODE (GEO accession number GSE40699)^42^ and an independent study profiling a number of different tissue types (Slieker *et al*), including fat cells^43^. In all cases, 450k DNAm profiles were corrected for type-2 probe bias using BMIQ^38^. In the case of the Slieker *et al* data, we verified that clustering samples over the top principal components, segregated samples according to tissue type. As there were multiple adipose tissue samples, we averaged these to define an initial reference adipose cell DNAm profile. To construct reference profiles for the deconvolution algorithm, we selected CpGs according to two criteria: (i) an absolute difference in beta-value between the HMEC profile and the averaged adipose one, >0.7, and (ii) that it mapped to a DNAse hypersensitive site, as these sites are more likely to represent tissue-specific markers^22^. This resulted in reference centroid DNAm profiles for HMEC and adipose tissue, defined over 1,320 CpGs. Given an independent sample with a 450k DNAm profile, we then estimated its proportional fat content using constrained projection (CP)^44^. Although stromal cells are also present in breast tissue, we here assume that this fraction is part of the estimated HMEC fraction. Thus, for any given sample, *w*_(HMEC)_*+w*_(FAT)_=1.

To validate our deconvolution algorithm, we collected Illumina 450k DNAm data for independent adipose tissue samples from the Stem Cell Matrix Compendium^45^. We also downloaded the ENCODE 450k DNAm data for a normal breast cell lines (MCF10A) and a breast cancer cell-line (MCF7). As before, all these data were corrected for type-2 probe bias using BMIQ. Weight estimates for each samples were then obtained using CP. R-code implementing CP is available under the filename ‘*InferPropCP.R*' as Supplementary Software 2.

### Demonstration of the stochasticity of DNAm field defects

For each hypervariable DVMC and normal-adjacent sample, we estimated a z-statistic, by comparing its DNAm beta-value to the average and standard deviation of the same CpG across the 50 normal samples from cancer-free women. From the z-statistics, one can then estimate a corresponding *P*-value. A binarized matrix was then constructed using a *P*-value threshold of 0.001, so that entries with *P*-values<0.001 were assigned a ‘1' (representing a methylation hit), entries with *P*-values>0.001 were assigned a ‘0'. This threshold corresponded roughly to a FDR of ∼0.05. For each DVMC, we then estimated its frequency of alteration across the 42 normal-adjacent samples. DVMCs that are more frequently altered than expected by random chance would indicate that these sites are altered in a non-stochastic manner. Thus, to assess this, we used a Monte-Carlo approach, whereby we scrambled up the binarized data matrix (4,062 hypervariable DMVCs x 42 samples with a total of 488,529 hits, that, 1's, representing 30% of entries in the matrix) and recomputed the expected null frequencies of alteration. Randomization was done multiple times and finally compared the maximal null frequencies with the maximal observed ones to assess whether any DVMCs exhibited more alterations than expected by chance.

### Comparison of the heterogeneity of DNAm patterns

We used a similar approach to the one described in the previous paragraph to assess the degree of heterogeneity of DNAm patterns in normal-adjacent tissue compared with breast cancer. Specifically, we used the same binarized deviation DNAm data matrix described above to estimate the fractional overlap of significant DNAm deviations (across all 4,062 hypervariable DVMCs) between every pair of normal-adjacent samples, as well as between every pair of matched breast cancers. So, this analysis was restricted to the 42 women who provided both a normal-adjacent and breast tumour specimen. In addition to the fractional overlap, we also estimated the Manhattan distance between every pair of normal-adjacent samples, as well as between every pair of matched breast cancers. To assess the statistical significance of the overlaps and distances, we used a Monte-Carlo approach in which we randomly permuted the DVMCs for each sample separately, recomputing the overlaps and distances. A total of 1,000 Monte-Carlo runs were performed. The average overlaps and distances were recorded for each run. The resulting null distributions were observed to be very tight, resulting in the comparison of the mean of these null distributions to the observed means for the observed (unpermuted) binary deviation matrices. We note that this Monte-Carlo approach is important in order to adjust for the inherently higher frequency of DNAm deviations exhibited by the breast cancer samples compared with normal-adjacent tissue.

### Functional epigenetic modules (FEM) algorithm and GSEA

To infer interactome modules that represent hotspots of differential DNA methylation, we used the interactome and procedure described by us previously^27^,^28^,^29^. Briefly, because we did not have matched mRNA expression data, we applied the version of FEM that only uses differential DNA methylation statistics (the EpiMod algorithm). Specifically, the weights in the interactome network were constructed from differential DNA methylation statistics between the 42 normal-adjacent samples and the 50 normal samples from healthy subjects. All other parameters of the FEM algorithm were run as done previously^27^,^28^,^29^. GSEA was performed using one-tailed Fisher exact tests using the Molecular Signatures Database^46^. Fisher-test *P*-values were corrected for multiple testing using the Benjamini Hochberg correction procedure.

### Construction of progression Z and personalized deviation scores

For each category of DVMCs (hypervariable+hypermethylated, hypervariable+hypomethylated, hypovariable+hypermethylated, hypovariable+hypomethylated) and for each sample, we constructed a progression Z-score, as the average Z-statistic over all CpGs in that category. Specifically, for a given CpG site in a category of DVMCs, we first computed the Z-statistic of its DNA methylation beta-value in sample *s* relative to the mean and standard deviation across all the 50 normal healthy samples. For the same sample *s*, we then averaged these Z-statistics over all CpGs within a given DVMC category. Thus, for the hypermethylated DVMC categories, larger positive Z-scores indicate average larger deviations from the normal state, whereas for the hypomethylated DVMC classes, larger negative Z-scores indicate stronger levels of hypomethylation relative to the normal state.

In addition, for the 42 matched breast cancers, we also computed an individualized deviation score as follows. Again, focusing on a specific category of DVMCs, we computed the average deviation in DNA methylation beta-value between the breast cancer sample and its corresponding matched normal-adjacent sample, the average being computed over all CpGs within the DVMC class. Once again, for the DVMC categories that are hypermethylated in normal-adjacent tissue compared with normal samples, we would expect positive deviation scores for those breast cancers that show further increases in DNA methylation compared with their matched normal adjacent sample; conversely, for the hypomethylated DVMCs, we would expect negative deviation scores for those breast cancers that show further decreases in DNA methylation compared with their matched normal-adjacent tissue.

### Coordination versus mutual exclusivity analysis

To assess if the DNA methylation changes between normal-adjacent and normal tissue, and within the inferred functional epigenetic modules (from the FEM/EpiMod analysis), are occurring in a coordinated or mutually exclusive fashion within a sample, we first constructed a binary deviation matrix over all genes in the module and all 42 normal-adjacent samples. This matrix was constructed from the *Z*-scores estimated previously, with *P*-values then estimated using a standard normal *N(0,1)* distribution. For *P*-values<0.05, a given entry in the binary matrix was assigned a value 1, otherwise a 0. The Manhattan distance was then estimated between all pairs of genes in the module with at least 10% significant deviations (that is, using only those rows/genes for which there were at least 4 (∼0.1 × 42) 1's). To assess statistical significance of the average Manhattan distance, we performed an independent permutation of samples for each row/gene, recomputing the average null Manhattan distance. A total of 1,000 Monte-Carlo randomization were performed to generate a null distribution for the average Manhattan distance.

### Validation of epigenetic field defects

We obtained Illumina 450k DNA methylation data for a cohort of 70 normal breast tissue samples collected adjacent to invasive breast cancers, as well as of 18 normal samples from reduction mammoplasty^18^. The series matrix file was downloaded from GEO (GSE67919) and processed with the same pipeline as the Erlangen set.

### Ductal carcinoma *in-situ* data sets

We obtained two separate DNA methylation data sets, described previously^20^,^21^. One data set profiled 15 normal-adjacent breast tissues and 40 DCIS (breast carcinomas) with Illumina Infinium 450k data, available from GEO (GSE66313)^20^. We downloaded the series matrix file and processed the 450k data using the same pipeline as for the primary Erlangen set. Another data set profiled 5 normal breast samples (from reduction mammoplasty surgeries) and 24 DCIS (breast carcinomas) using RRBS. We downloaded the normalized methylation fraction data matrix for 414,930 loci with at least 20-fold coverage, available from GEO (GSE69994)^21^. Illumina 450k probes were mapped to the RRBS data using a 200-bp window around the 450k probes: that is, methylation fractions for RRBS loci within 100 bp on either side of an Illumina 450k probe were averaged.

## Authors' contributions

Study was conceived by M.W. Study design was done by M.W. and A.E.T. Statistical analysis was performed by A.E.T. with assistance from G.Y. Manuscript was written by A.E.T. and M.W. A.J contributed experimental work. P.A.F., M.R., M.B. contributed samples for analysis.

## Additional information

**Accession codes:** The microarray data have been deposited in the GEO database under accession code GSE69914.

**How to cite this article:** Teschendorff, A. E. *et al*. DNA methylation outliers in normal breast tissue identify field defects that are enriched in cancer. *Nat. Commun*. 7:10478 doi: 10.1038/ncomms10478 (2016).

## Supplementary Material

Supplementary Information Supplementary Figures 1-17, Supplementary Tables 1-9 and Supplementary References.

Supplementary Data 1 All 7318 differentially variable and differentially methylated CpGs (DVMCs). Columns label Illumina Probe ID, t-statistic of differential methylation, its P-value, the log2 ratio of the variances in normal-adjacent to normal samples, its corresponding Bartlett test P-value and Q-value.

Supplementary Data 2 Transcription factor binding site enrichment analysis. Odds Ratio (OR) and one-tailed Fisher-test P-values of enrichment of 58 TF ChIP-Seq binding sites, as determined in hESCs, among the 4 classes of DVMCs: hypervariable + hypermethylated (dvUPdmUP), hypervariable + hypomethylated (dvUPdmDN), hypovariable+hypermethylated (dvDNdmUP) and hypovariable+hypomethylated (dvDNdmDN)

Supplementary Data 3 Gene Set Enrichment Analysis of DVMCs against the Molecular Signatures Database. GSEA results for each class of DVMCs, hypervariable+hypermethylated (dvUPdmUP), hypervariable+hypomethylated (dvUPdmDN), hypovariable+hypermethylated (dvDNdmUP) and hypovariable+hypomethylated (dvDNdmDN). Columns label gene list, number of genes in list, number of genes present, fractional representation, the number that overlap with DVMCs (mapped to unique genes), the Odds Ratio (OR), the Fisher-test P-value (one-tailed), the adjusted P-value (Benjamini-Hochberg) and the symbols of the enriched genes.

Supplementary Data 4 Sheet-1: Summary of all inferred EpiMods. This lists the entrez gene ID and symbol of the seed gene, the size of module, its modularity, its empirical Monte-Carlo derived P-value and the genes making up the module. Those highlighted in yellow are among the most significant ones, those in orange are the largest ones. Sheet-2: Full listing of inferred EpiMods. For each EpiMod this lists the entrez gene ID, gene symbol, t-statistic of differential DNA methylation, associated P-value and statistic value used in algorithm.

Supplementary Software 1 An R-script to perform feature selection on a large omic data set using the iEVORA algorithm. The algorithm is aimed at large DNA methylation data sets, where one may wish to find features (mostly CpGs) which differ between two normal cellular phenotypes, but with one of these phenotypes representing cells which are at risk of neoplastic transformation. The algorithm uses a differential variability (DV) step to increase power/sensitivity, but uses a standard t-test to rank significant DV CpGs (DVCs). This last step is done to regularize the DV-test, which is overly sensitive to single outliers. The output of the script is a list of top ranked differentially variable and differentially methylated CpGs (DVMCs). Further details are found in the script itself.

Supplementary Software 2 R-script InferPropCP.R. An R-script to infer the proportions of a priori known cell subtypes present in a sample representing a mixture of such cell-types. Inference proceeds via constrained projection (CP) and quadratic programming. The output of the script is the inferred proportions of cell subtypes within the input sample provided. Further details are found in the script itself.
