# Supplementary material for: DNA methylation outliers in normal breast tissue identify field defects that are enriched in cancer
Source: Nat Commun. 2016 Jan 29;7:10478. doi: 10.1038/ncomms10478 (PMC4740178; doi:10.1038/ncomms10478)
Supplement: Supplementary Information — Supplementary Figures 1-17, Supplementary Tables 1-9 and Supplementary References. [file ncomms10478-s1.pdf]

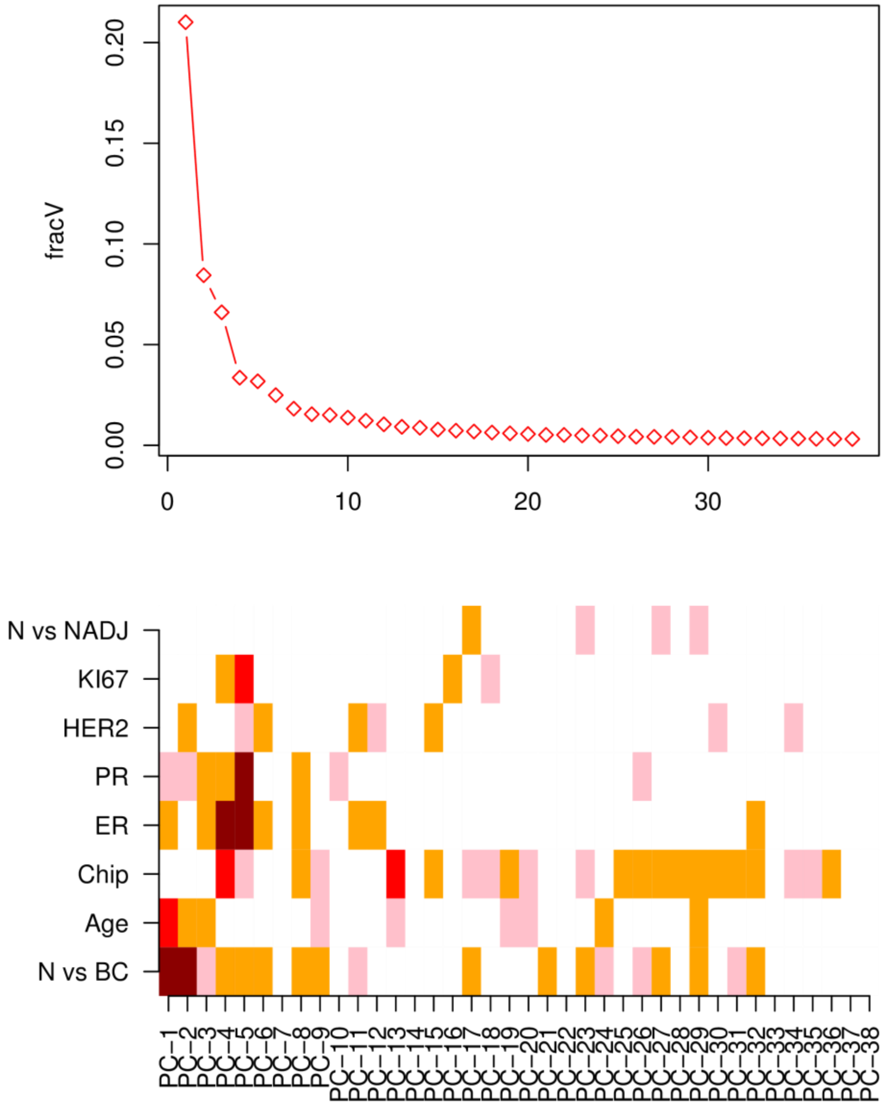

Supplementary Figure 1: SVD analysis. Top panel: Fraction of variation of the data explained by the top 38 singular vectors of a Singular Value Decomposition (SVD) on the Erlangen 450k DNAm data set. RMT [1] estimated 38 significant singular vectors/principal components. **Bottom panel:** Heatmap of association P-values between the 38 PCs and various biological and technical factors, including Normal versus Breast Cancer, Age, Beadchip, Estrogen receptor status (ER), Progesterone receptor status (PR), HER2 status, and proliferation KI67 index, as well as Normal vs Normal-Adjacent. Color codes for P-values: P<1e-10 (brown), P<1e-5 (red), P<1e-3 (orange), P<0.05 (pink), P > 0.05 (white). P-values were obtained using a linear ANOVA model.

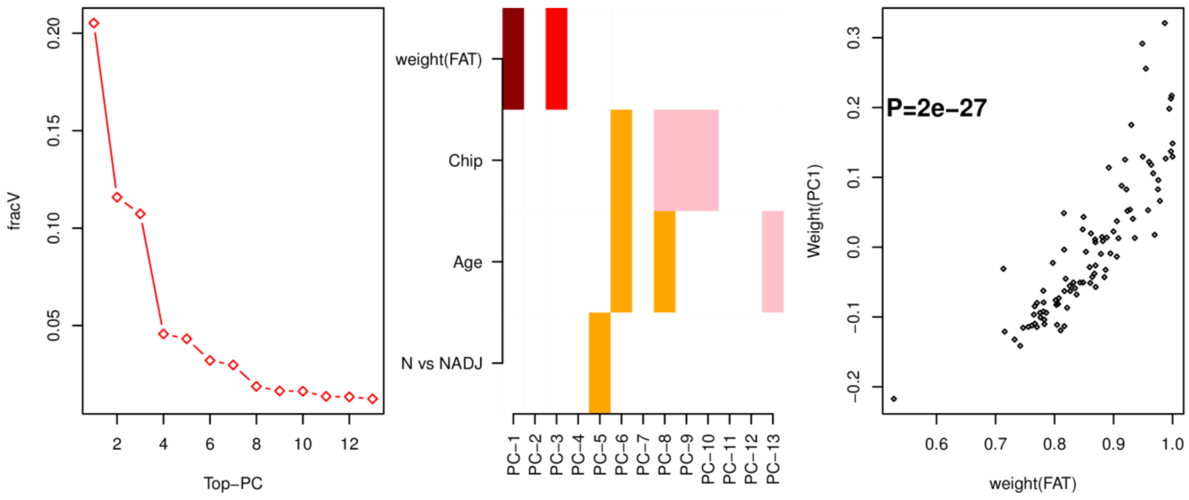

Supplementary Figure 2: SVD and fat-content analysis. Left panel: Fraction of variation of the data explained by the top 13 singular vectors of a SVD on the Erlangen 450k DNAm data set, restricted to the 92 normal samples (50 from healthy subjects, 42 from breast cancer patients). RMT [1] estimated 13 significant singular vectors/principal components. **Middle panel:** Heatmap of association P-values between the 13 PCs and various biological and technical factors, including Normal versus Normal-Adjacent (N vs NADJ), Age, Beadchip and the estimated fraction of adipose (fat) cells – weight(FAT). Color codes for P-values: P<1e-10 (brown), P<1e-5 (red), P<1e-3 (orange), P<0.05 (pink), P > 0.05 (white). P-values were obtained using a linear ANOVA model. **Right panel:** Scatterplot of the weights of the top PC (y-axis) against the estimated fraction of fat cells (x-axis). P-value is from a linear regression.

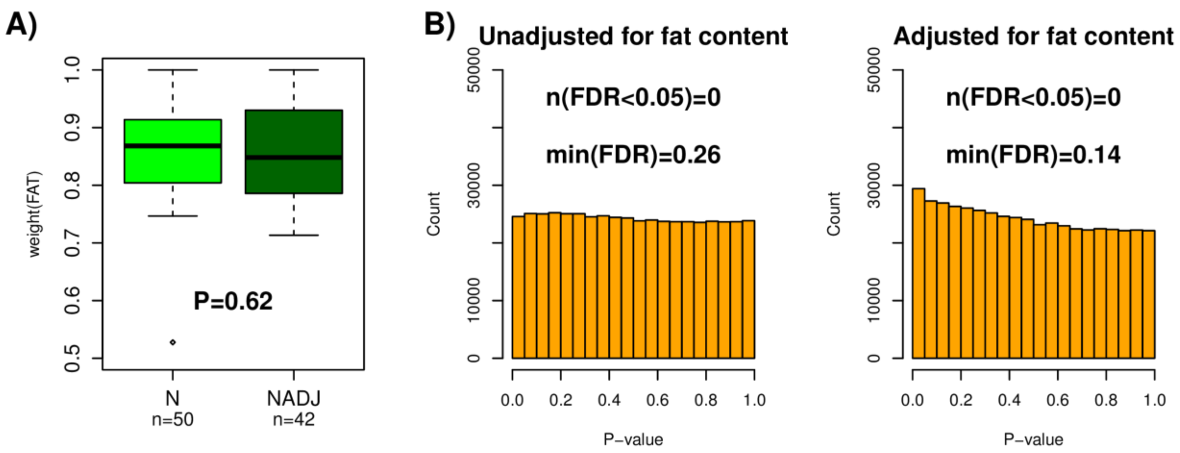

Supplementary Figure 3: Effect of fat content adjustment on supervised analysis using t-tests. A) Boxplots of the estimated fraction of adipose cells in the normal and normal-adjacent samples. Horizontal black lines indicate the median, the upper and lower hinges define the upper and lower quartiles, and the whiskers extend to 1.5 times the inter-quartile range. P-value is from a two-sided Wilcoxon rank sum test. **B)** Histograms of t-test P-values (adjusted and unadjusted for adipose/fat content) testing for differential DNA methylation between normal and normal-adjacent samples. In each case, we give the minimum false discovery rate (FDR) and the number of probes passing an FDR < 0.05.

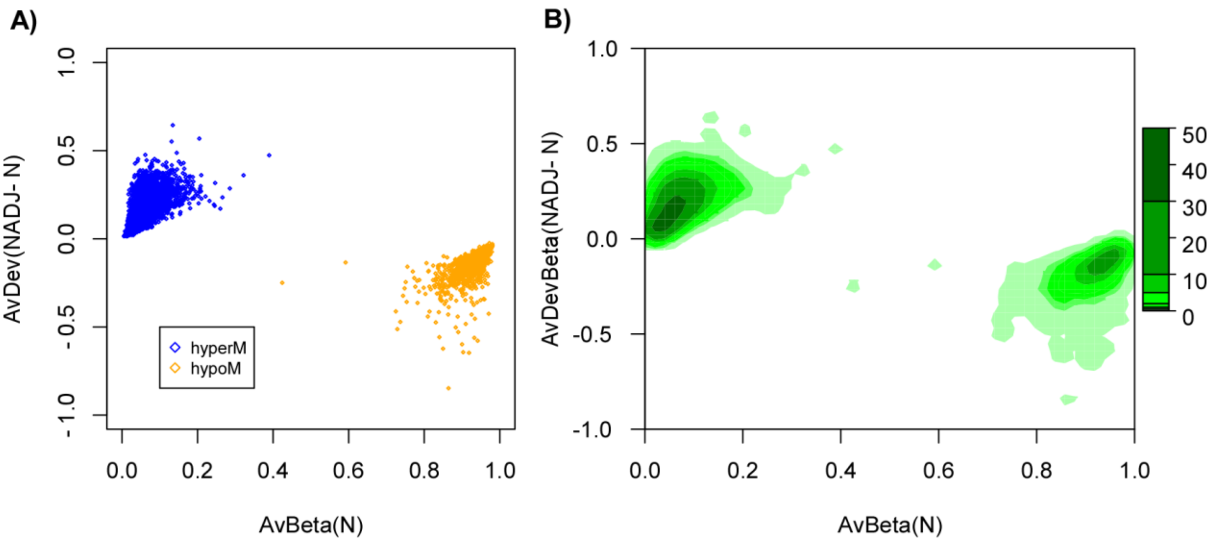

Supplementary Figure 4: Average deviations in DNA methylation of field defects against mean DNA methylation levels in normal tissue. A) Scatterplot of the average deviation in DNAm beta-value between the 42 normal-adjacent samples and the 50 normal samples (y-axis: AvDev(NADJ-N)) versus the average DNAm beta-value across the 50 normal samples (x-axis: AvBeta(N)) for all 4062 hypervariable DVMCs. Blue indicates those that are hypermethylated, orange those that are hypomethylated in NADJ samples compared to normals (N). **B)** Exactly the same as A), but now using a density representation, showing that most of the hypervariable-hypermethylated DVMCs are characterised by DNAm deviations from the normal state on the order of 20-30% in beta-value scale. For the case of hypervariable-hypomethylated DVMCs, the absolute deviations are marginally smaller, but most deviations are on the order of 20% decreases in DNAm levels.

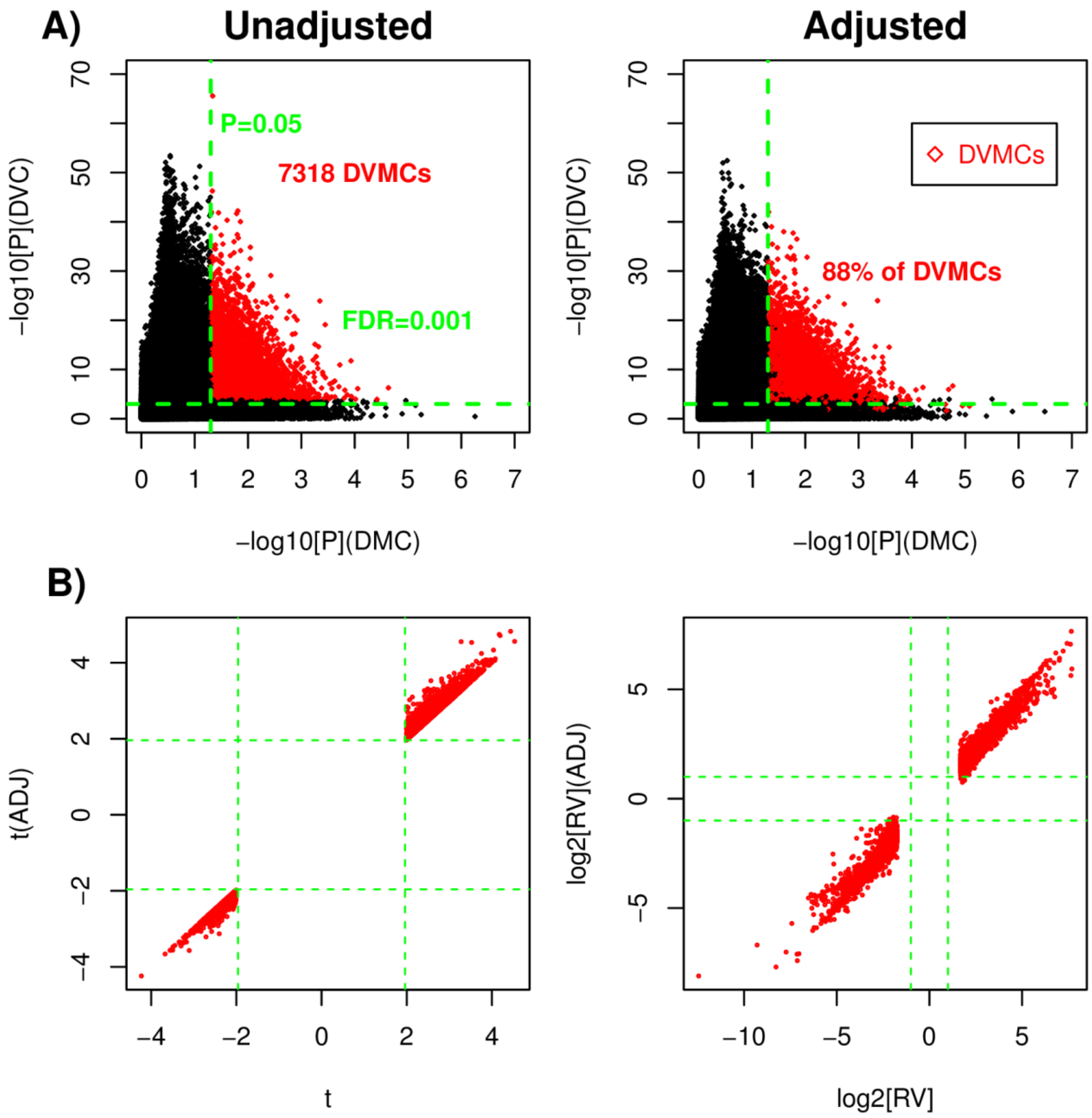

Supplementary Figure 5: Definition of DVMCs and their robustness to corrections for adipose cell content. A) Scatterplot of the $-\log_{10}$[P-values] of t-tests (testing differential means) (x-axis) against $-\log_{10}$[P-values] from Bartlett's test (testing differential variability) (y-axis) in the unadjusted case (left panel), as well as in the case for adjustment for a sample's fat content (right panel). The green dashed lines in the left panel define the thresholds used to define differentially variable (FDR<0.001) differential methylated (uncorrected P<0.05) CpGs (DVMCs), indicated in red. The right panel shows the same DVMCs in red. Observe how most

of the DVMCs (88%), as defined in the left panel, remain significant after adjustment for adipose content. **B)** Left panel: scatterplots of the t-statistics in the unadjusted (x-axis) vs adjusted (y-axis) analysis for the 7318 DVMCs. Right panel shows the corresponding log_2-ratio of the variances in the unadjusted (x-axis) vs adjusted (y-axis) analysis, where log_2[RV]=log_2[Variance in NADJ / Variance in N] . Green dashed lines indicate the lines of P=0.05.

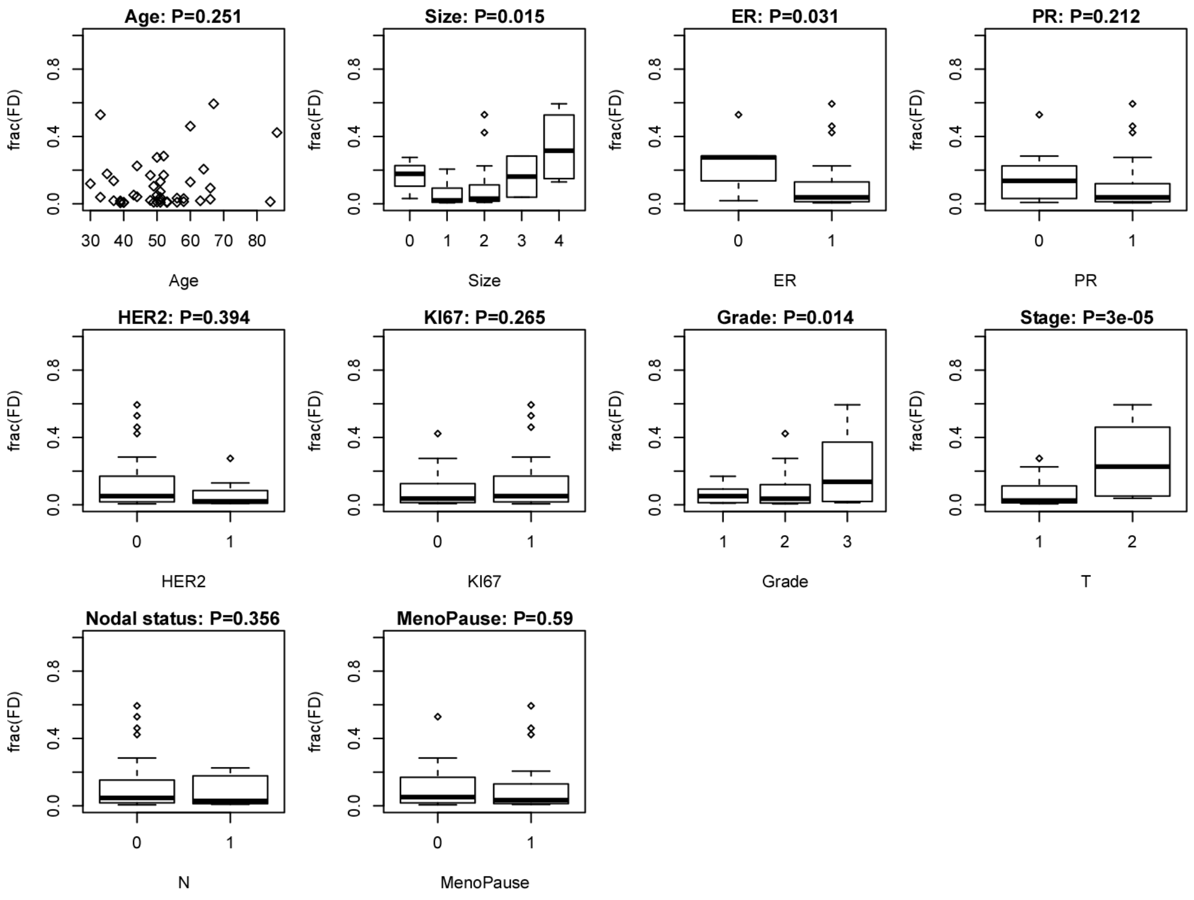

Supplementary Figure 6: Association of field defects with breast cancer phenotypes. Correlation of the fraction of epigenetic alterations (significant DNAm deviations) at hypervariable DVMC loci (frac(FD): y-axis) in the 42 normal-adjacent samples with 10 different clinical or demographic characteristics of the matched breast cancers. For all factors, P-value was estimated using a linear model.

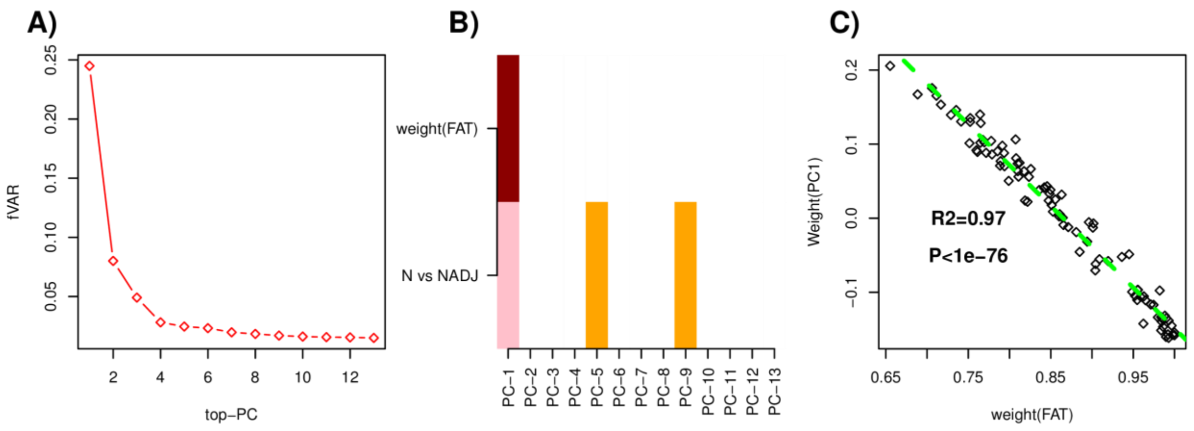

Supplementary Figure 7: SVD and fat content analysis in the validation set. A) Fraction of variation of the data explained by the top 13 singular vectors of a SVD on the validation 450k DNAm data set, consisting of 18 normal samples (from reduction mammoplasty of healthy subjects, N) and 70 normal samples adjacent to invasive breast cancers (NADJ) [2]. RMT [1] also estimated 13 significant singular vectors/principal components in this data set. **B)** Corresponding heatmap of association P-values between the 13 PCs and either N vs NADJ or the estimated fraction of fat cells (weight(FAT)). Color codes for P-values: P<1e-10 (brown), P<1e-5 (red), P<1e-3 (orange), P<0.05 (pink), P > 0.05 (white). P-values were obtained using a linear ANOVA model. **C)** Scatterplot of the weights of the top PC (y-axis) against the estimated fraction of fat cells (x-axis). P-value and R^2 values are from a linear regression.

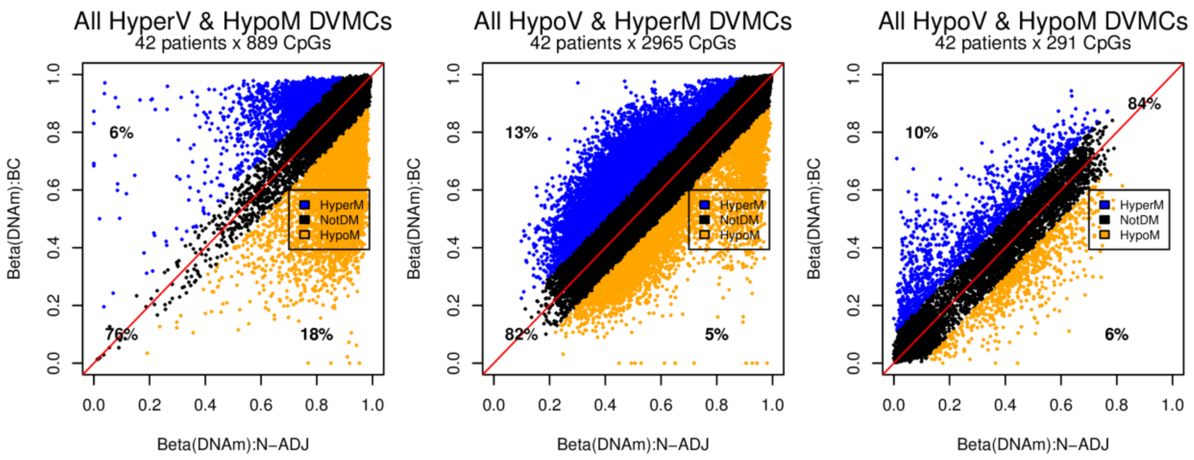

Supplementary Figure 8: Patterns of DNA methylation progression for the three classes of DVMCs. Scatterplots of DNA methylation for three categories of DVMCs (as indicated above each plot) and restricting to the 42 matched normal-tumour pairs, with x-axis labelling the beta-value in the normal adjacent sample, and y-axis labelling the corresponding beta-value in the matched breast tumour. Blue and orange points represent DVMCs in breast cancer patients for which the change in mean DNA methylation between normal-adjacent and cancer was larger or lower than 0.1 in absolute terms, with blue indicating hypermethylation and orange hypomethylation. Black data points represent DNAm changes less than 0.1 in absolute terms. The proportion of data points which are hypermethylated, hypomethylated and which do not exhibit at least 0.1 changes are indicated.

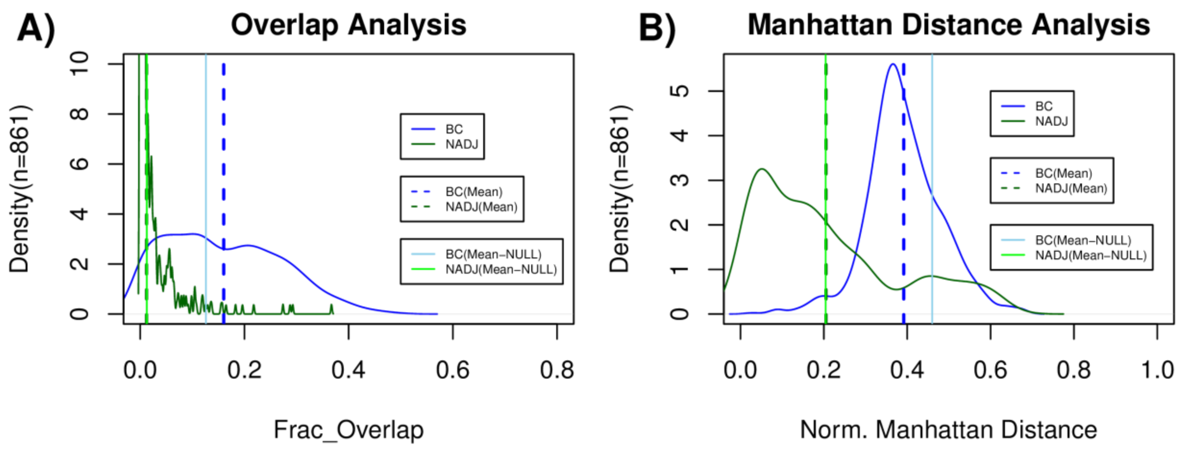

Supplementary Figure 9: Pairs of breast cancers are more similar across DVMCs than their corresponding pairs of normal-adjacent tissue. A) For each pair of normal-adjacent (NADJ, n=42) or breast cancer samples (BC, n=42), we estimate the fractional overlap of hypervariable DVMCs which exhibit significant DNAm deviations from the normal state. There are thus 42*41*0.5=861 data points in each density curve. Superimposed on the plot we also provide the mean of the two density distributions (dashed vertical lines). In order to assess statistical significance we also compare these means to the means of the null distribution obtained by 1000 Monte Carlo randomisations in which DVMCs were randomly permuted (separate permutation for each of the 42 samples) (continuous vertical lines). This Monte-Carlo analysis adjusts for the fact that breast cancers naturally exhibit higher fractions of DNAm deviation than normal-adjacent samples. We note that the null distribution is very tight with extremely low variance, hence it is shown as a vertical line indicating the mean. **B)** Exactly as A), but for a distance measure based on the Manhattan metric.

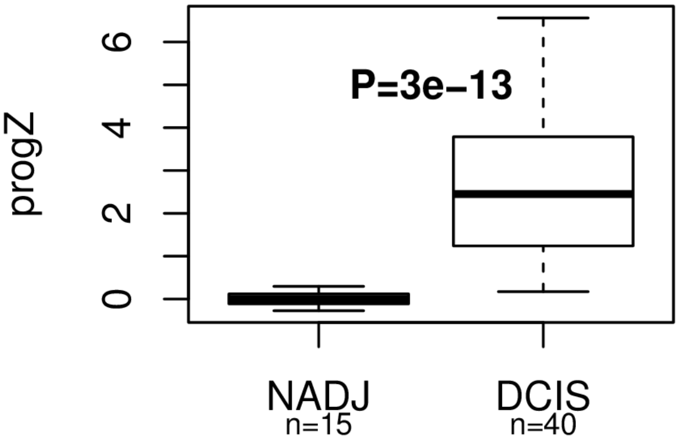

Supplementary Figure 10: Validation of field defects in DCIS samples. Progression Z-scores, defined over the 3173 hypervariable and hypermethylated DVMCs from the Erlangen discovery cohort, as computed in the Illumina 450k DNAm data set of Johnson et al [3]. Boxplots compare the progression Z-scores between ductal in-situ breast carcinomas (DCIS) to normal-adjacent tissue (NADJ). Number of samples in each group are indicated. P-value is from a Wilcoxon-rank sum test, indicating that DCIS samples exhibit substantial increased deviations in DNAm at the 3173 sites compared to NADJ samples.

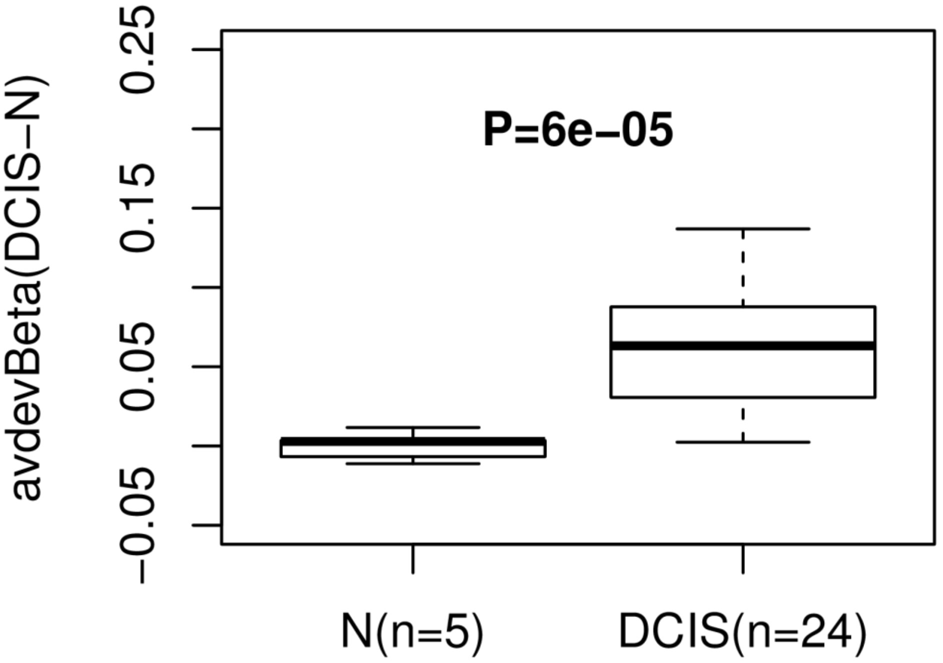

Supplementary Figure 11: Validation of field defects using RRBS data of DCIS samples Boxplots of the average deviation in beta-valued DNA methylation between 24 DCIS and 5 normal mammoplasty reduction samples, for the reduced-representation bi-sulfite sequencing (RRBS) data from Abba et al [4]. The average deviations were estimated from 983 loci with 20-fold coverage in the RRBS data and mapping within 100bp from one of the 3173 hypervariable and hypermethylated DVMCs identified in the Erlangen set. P-value is from a Wilcoxon rank sum test. Observe how these DVMCs also exhibit increased DNAm levels in the DCIS relative to the normals.

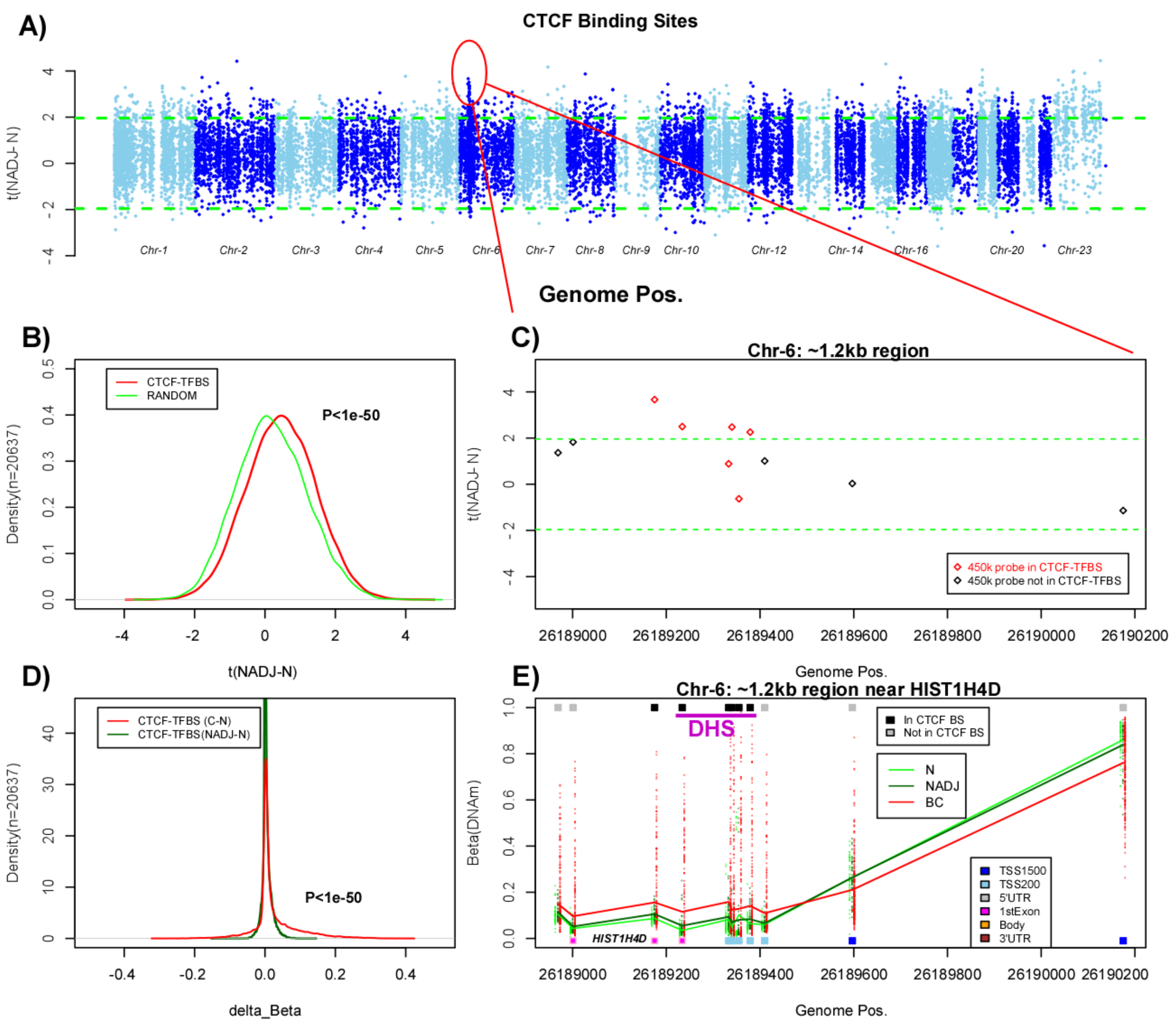

Supplementary Figure 12: Enrichment of CTCF TF binding sites among DNA methylation field defects. A) Manhattan type plot of the t-statistics of differential DNA methylation between normal adjacent (NADJ) and normal breast tissue (N) of 450k probes mapping to CTCF binding sites (20637 sites). Green dashed-lines represent the lines corresponding to P=0.05. **B)** Density distribution of the t-statistics of differential methylation between normal adjacent (NADJ) and normal breast tissue (N) of 450k probes mapping to CTCF binding sites (red) compared to a randomly chosen set (green). P-value is from a Wilcoxon rank sum test. **C)** Zoomed in version of A) focusing on a 1.2kb region on chromosome-6, but now showing all 450k probes in the region, with those mapping to CTCF binding sites indicated in red. **D)** Comparison of the density distribution of average differences in DNA methylation (delta_Beta) for the 20637 probes mapping to CTCF binding sites (CTCF TFBS) between cancer and normal adjacent tissue (C-N),

to the corresponding differences between normal adjacent and normal tissue (NADJ-N). P-value is from a Wilcoxon rank sum test. **E)** As C) but now showing the DNA methylation beta values for all samples and probes the region, which maps to *HIST1H4D*. The horizontal lines represent the mean DNAm values in each group: normal (N), normal-adjacent (N-ADJ) and breast cancer (BC). Probes/CpGs have been annotated according to whether they fall in a CTCF binding site, and which gene region they map to, and also if they constitute DNase Hypersensitive Sites (DHSs).

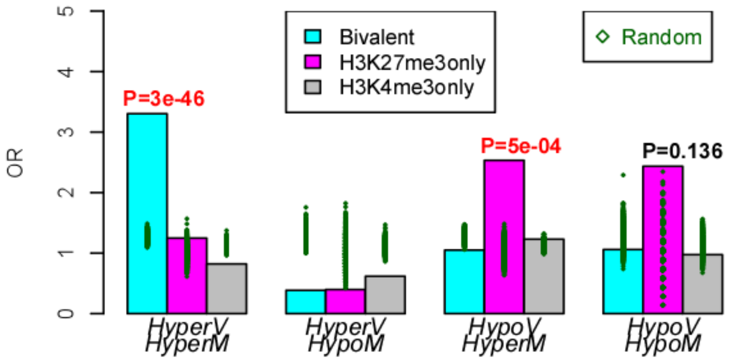

Supplementary Figure 13: Enrichment analysis of DVMCs. Odds Ratio (OR) of enrichment of 3 classes of genes (Bivalent, H3K27me3 only, H3K4me3 only marked genes in hESCs) among the four classes of DVMCs: hypervariable and hypermethylated, hypervariable and hypomethylated, hypovariable and hypermethylated, hypovariable and hypomethylated. P-values were estimated using a one-tailed Fisher exact test and in red we show the significant enrichment ORs. In green, we show the ORs expected for a random selection of CpGs matched for size and gene regulatory region (1000 random samplings).

FGFR1-EpiMod

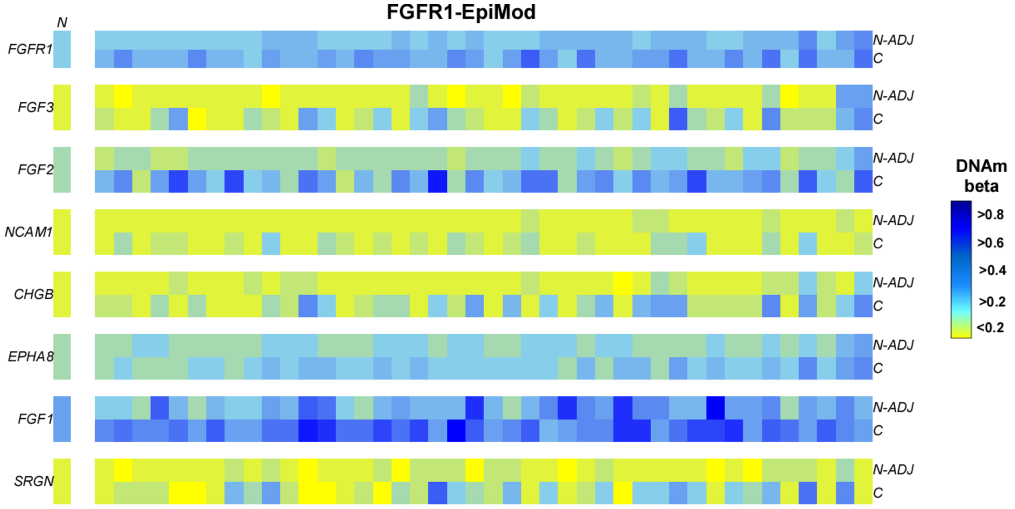

FOXA2-EpiMod

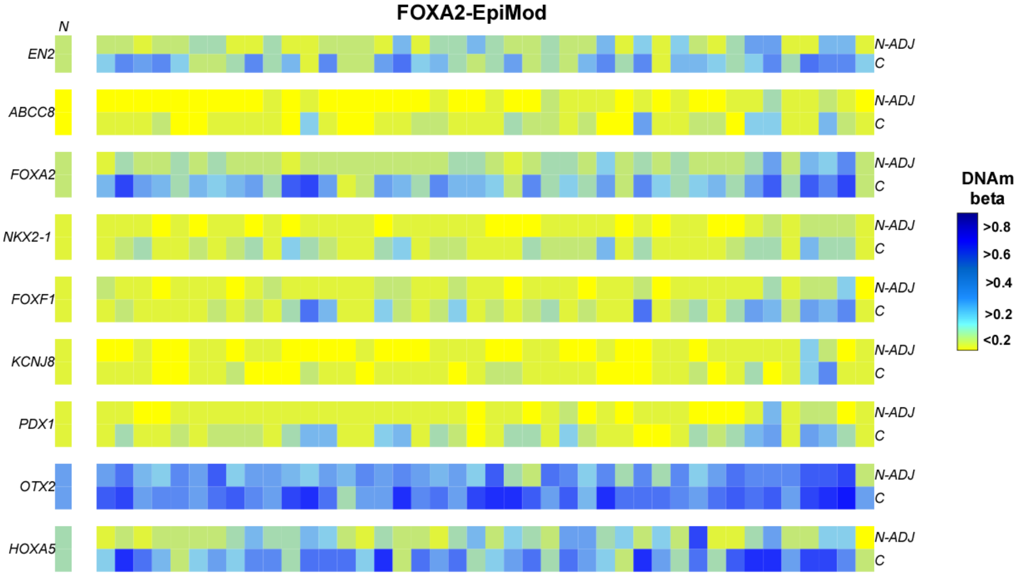

PAX6-EpiMod

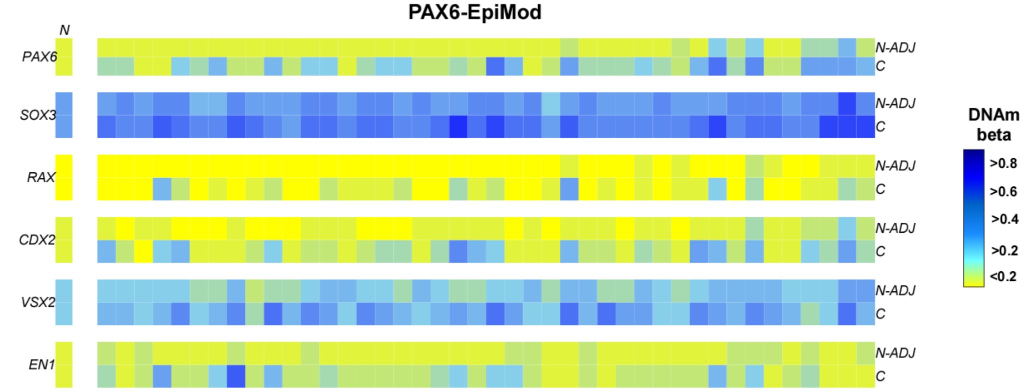

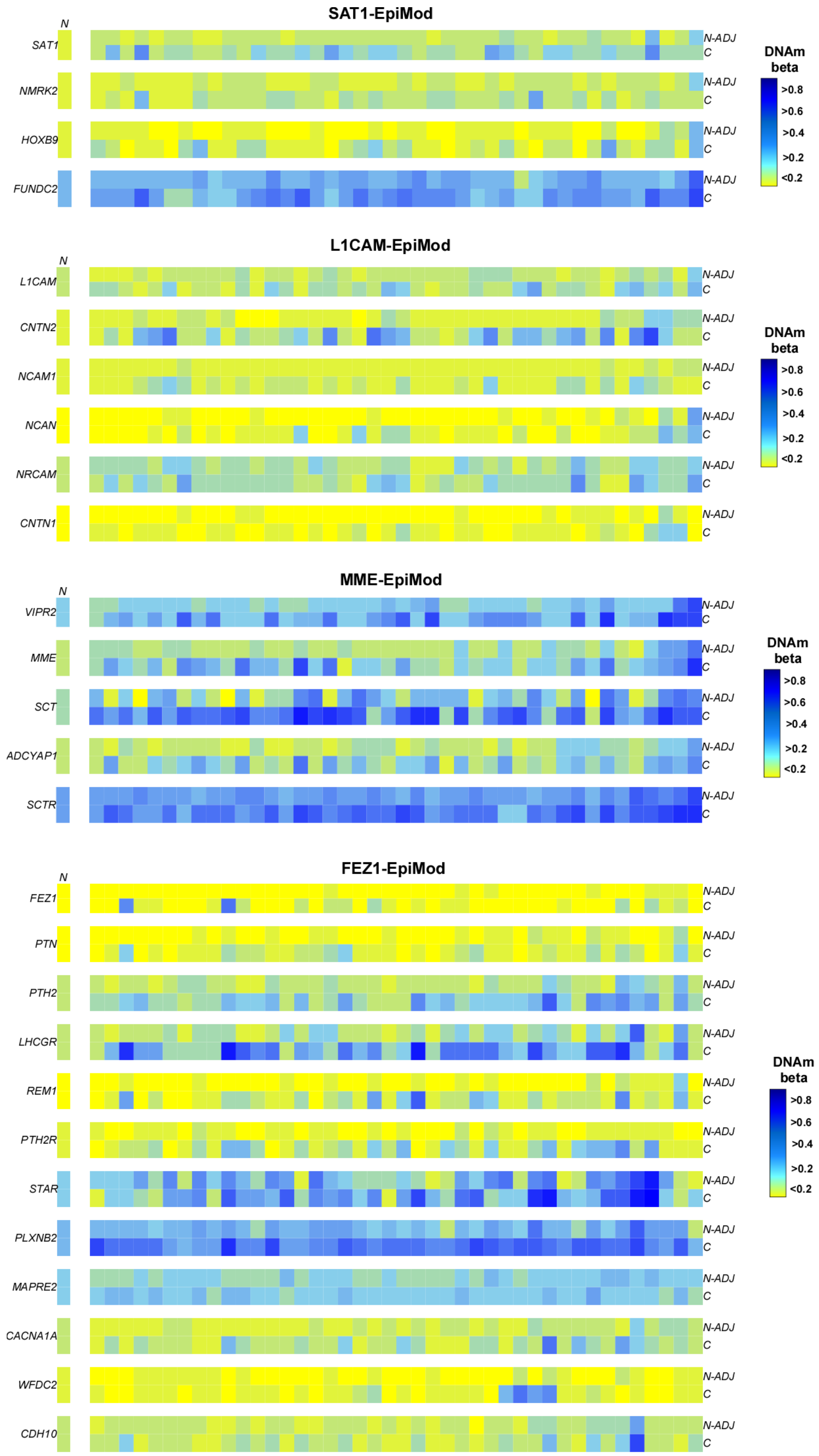
SAT1-EpiMod
N
SAT1
NMRK2
HOXB9
FUNDC2
N-ADJ
C
DNAm beta
>0.8
>0.6
>0.4
>0.2
<0.2
L1CAM-EpiMod
N
L1CAM
CNTN2
NCAM1
NCAN
NRCAM
CNTN1
N-ADJ
C
DNAm beta
>0.8
>0.6
>0.4
>0.2
<0.2
MME-EpiMod
N
VIPR2
MME
SCT
ADCYAP1
SCTR
N-ADJ
C
DNAm beta
>0.8
>0.6
>0.4
>0.2
<0.2
FEZ1-EpiMod
N
FEZ1
PTN
PTH2
LHCGR
REM1
PTH2R
STAR
PLXNB2
MAPRE2
CACNA1A
WFDC2
CDH10
N-ADJ
C
DNAm beta
>0.8
>0.6
>0.4
>0.2
<0.2

Supplementary Figure 14: Heatmaps of DNA methylation of EpiMod members. We show heatmaps for 7 EpiMods (defined by seed genes, *FGFR1*, *FOXA2*, *PAX6*, *SAT1*, *L1CAM*, *MME* and *FEZ1)*, and across the 42 matched normal-adjacent (N-ADJ) breast cancer (BC) pairs. In the case of the normal samples from healthy subjects (N) we show the average DNA methylation values across all 50 samples.

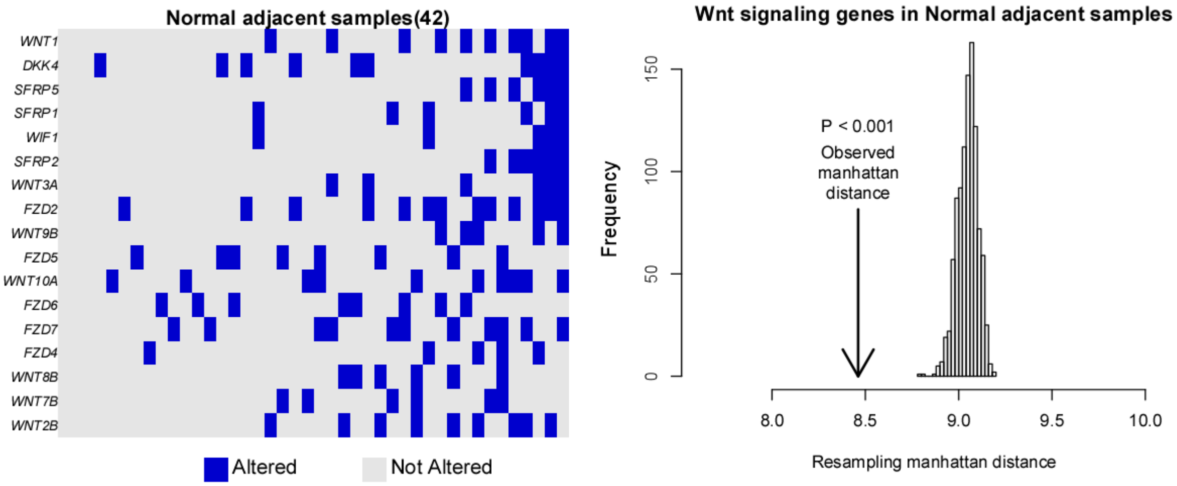

Supplementary Figure 15: Field defects exhibit coordinated DNA methylation changes. Focusing on WNT signalling pathway members which showed significant DNAm alterations in at least 10% of the 42 normal-adjacent samples compared to the 50 normal-healthy ones. Left panel is a binary matrix depiction of the DNAm alterations. A DNAm change in a normal-adjacent samples was considered altered if the P-value of its Z-statistic (as measured relative to the average and standard deviation over the 50 normal healthy samples) was less than 0.05. Right panel compares the observed Manhattan distance (the average distance over all gene-pairs) to the average distance obtained by permuting the columns of the matrix, independently for each row. P-value was derived from 1000 Monte Carlo randomisations.

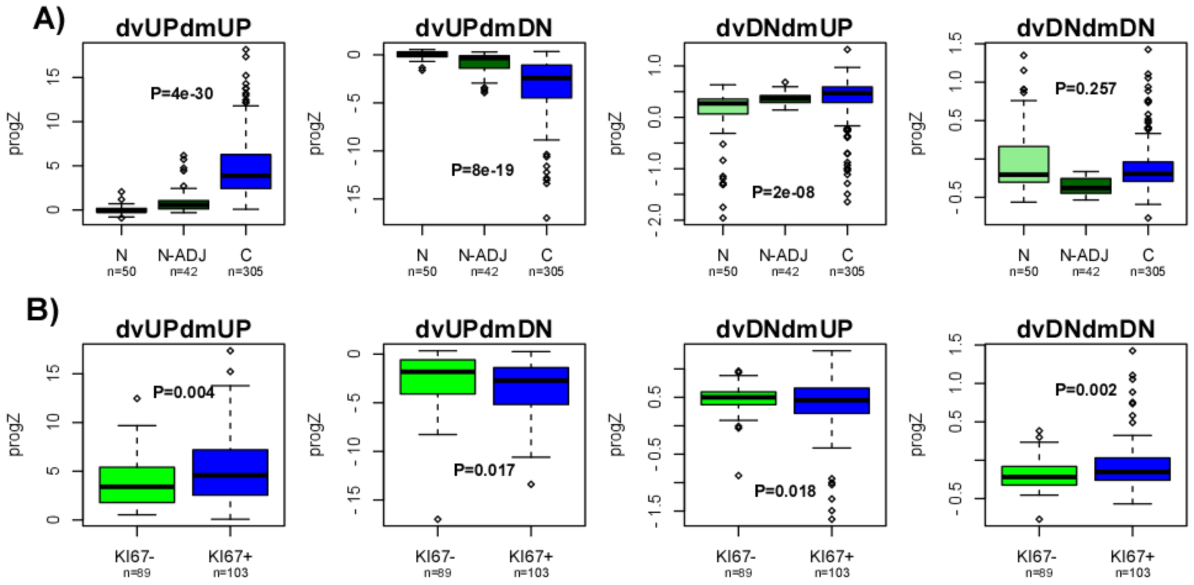

Supplementary Figure 16: Patterns of progression of DVMCs. A) Boxplots of progression Z-scores against sample status (N=normal-healthy, NADJ=normal adjacent, C=breast cancer) for each class of DVMC. P-values are from a linear regression. **B)** Boxplots of the same progression Z-scores against the proliferation index (KI67) for each class of DVMC. P-values are from a Wilcoxon-rank sum test.

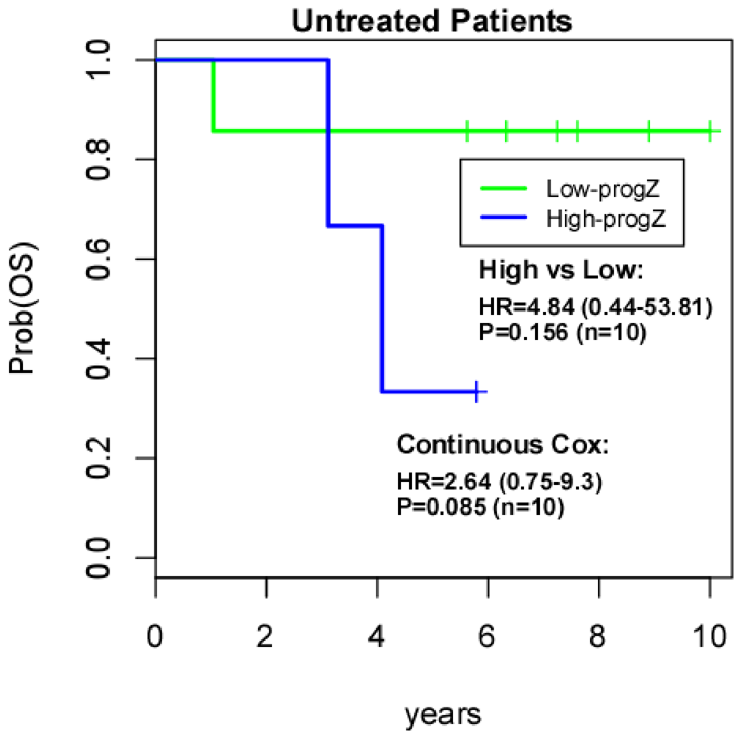

Supplementary Figure 17: Survival analysis for untreated subset of patients. Kaplan-Meier Survival Analysis of the untreated patients stratified according to low and high progression Z-scores. The two groups were defined exactly as in the Erlangen data set, i.e. by applying a partitioning around medoids (pam) clustering algorithm (with k=2) to the scores. The scores themselves were computed using only the hypervariable & hypermethylated DVMCs. We provide two Hazard Ratios (HR), 95%CI and P-values, one for the two group comparison and the other for the Cox-regression against the (not binarised) progression Z-score. P-values are from a log-rank test.

	Healthy (n=50)	BC with NADJ (n=42)	BC without NADJ (n=263)
**Age**			
**Mean**	**49.5**	**51.1**	**61.7**
**SD**	**14.4**	**12.2**	**12.3**
**Wilcox-test P-values:**			
**Healthy vs. BC(+NADJ)**	**0.91**		
**Healthy vs BC (noNADJ)**	**1.00E-07**		
**BC (NADJ) vs BC (noNADJ)**	**5.00E-07**		
**Ethnicity**			
**White Caucasian**	**50 (100%)**	**41 (98%)**	**263 (100%)**
**South-East Asian**	**0 (0%)**	**1 (2%)**	**0 (0%)**

Supplementary Table 1: Demographic details (Age and Ethnicity of women in the cohort). Details have been stratified according to 3 groups: healthy cancer-free women (n=50), women with breast cancer (BC) who contributed a normal-adjacent (NADJ) sample (n=42) and women with breast cancer who did not contribute a normal-adjacent sample (n=263). Healthy cancer-free women who donated normal/non-neoplastic tissue had breast surgery due to a fibroadenoma (n=11), mastopathy (n=18), makromastia (n=2), suspected breast lesion (n=10) and other benign conditions (n=9).

		BC with NADJ (n=42)	BC without NADJ (n=263)	Fisher-test P
**ER**				
	**ER+**	**37 (88%)**	**217 (83%)**	
	**ER-**	**5 (12%)**	**43 (17%)**	
	**NA**	**0**	**3**	**0.65**
**PR**				
	**PR+**	**33 (79%)**	**184 (72%)**	
	**PR-**	**9 (21%)**	**72 (28%)**	
	**NA**	**0**	**7**	**0.46**
**HER2**				
	**HER2+**	**7 (17%)**	**36 (16%)**	
	**HER2-**	**35 (83%)**	**189 (84%)**	
	**NA**	**0**	**38**	**1**
**Grade**				
	**G1**	**5 (12%)**	**36 (14%)**	
	**G2**	**26 (62%)**	**125 (48%)**	
	**G3**	**11 (26%)**	**98 (38%)**	
	**NA**	**0**	**4**	**0.26**
**Stage**				
	**1**	**32 (76%)**	**154 (61%)**	
	**2**	**10 (24%)**	**82 (32%)**	
	**3**	**0 (0%)**	**10 (4%)**	
	**4**	**0 (0%)**	**8 (3%)**	
	**NA/other**	**0**	**9**	**0.24**
**Nodal Status**				
	**Negative**	**32 (76%)**	**210 (81%)**	
	**Positive**	**10 (24%)**	**48 (19%)**	
	**NA**	**0**	**5**	**0.41**
**Survival**				
	**Alive**	**38 (90%)**	**227 (86%)**	
	**Dead**	**4 (10%)**	**36 (14%)**	
	**NA**	**0**	**0**	**0.62**
**Histology**				
	**Ductal Adenocarcinoma**	**39 (93%)**	**201 (76%)**	
	**Lobular Carcinoma (NOS)**	**2 (5%)**	**29 (11%)**	
	**Infil Lobular Mixed**	**1 (2%)**	**1 (<1%)**	
	**Other**	**0 (0%)**	**32 (~13%)**	**P=0.01**

Supplementary Table 2: Table of clinical characteristics of breast cancers. Clinical characteristics of the breast cancers (BC) of women who contributed a normal-adjacent (NADJ) sample and those who did not contribute a normal-adjacent sample. P-values were evaluated using a two-tailed Fisher's exact test. In the case of low counts, 2000 permutations were used to estimate P-values.

CP weight estimates	Adipose tissue sample 1 (SCM)	Adipose tissue sample 2 (SCM)	MCF10A	MCF7
**Adipose cells**	1	1	0.18	0.49
**HMECs**	0	0	0.82	0.51

Supplementary Table 3: Validation of CP algorithm. Estimates of adipose cell content of two independent adipose tissue samples from the Stem-Cell Matrix Compendium (SCM) [5]. Also shown are the estimates of adipose cell content and HMECs for a normal breast cell line (MCF10A) and a ER+ breast cancer cell line (MCF7) using Illumina 450k DNAm data from ENCODE. Weight estimates were obtained using a constrained projection (CP) method as described previously.

DVMC class	Total	Mapped	TSS1500	TSS200	5'UTR	1st Exon	Body	3'UTR
dvUPdmUP	3173	1889	561 (30%)	421 (22%)	178 (9%)	126 (7%)	580 (31%)	23 (1%)
dvUPdmDN	889	507	84 (17%)	16 (3%)	33 (7%)	8 (2%)	335 (66%)	31 (6%)
dvDNdmUP	2965	1930	308 (16%)	349 (18%)	145 (8%)	85 (4%)	935 (48%)	108 (6%)
dvDNdmDN	291	174	33 (15%)	26 (15%)	37 (21%)	8 (5%)	63 (36%)	7 (4%)

Supplementary Table 4: Distribution of DVMCs according to gene region. Table shows how the differentially variable and differentially methylated CpGs (DVMCs) between normal-adjacent and normal breast tissue are distributed according to the directionality of differential variability and differential methylation change (e.g. dvUPdmUP means hypervariable and hypermethylated in normal-adjacent tissue compared to normal, whereas dvDNdmDN means hypovariable and hypomethylated in normal-adjacent tissue compared to normal-healthy). For those DVMCs which could be unambiguously mapped to a gene regional class (TSS1500, TSS200, 5'UTR, 1st Exon, Gene Body and 3'UTR), we show their distribution among these classes.

	progZ dvUPdmUP	progZ dvUPdmDN	progZ dvDNdmUP	progZ dvDNdmDN
**ER**	0.907	0.794	0.554	0.32
**PR**	0.068	0.403	0.536	0.271
**HER2**	0.021	0.13	0.005	0.125
**KI67**	0.004	0.017	0.018	0.002
**Death/Alive**	0.042	0.754	0.989	0.338
**Tumor Size**	0.005	0.08	0.498	0.22
**Patient Age**	0.053	0.047	0.823	0.602

Supplementary Table 5: Association of progression of DVMCs with clinical phenotypes. P-values of association (obtained using linear regression models) of clinical characteristics of the breast cancers (n=305) with the progression Z-scores for the four different classes of DVMCs, as indicated.

**Univariate**						
**progZ**	**LI**	**HR**	**HI**	**z**	**P**	**n**
dvUPdmUP	1.1	1.42	1.84	2.69	0.007	305
dvUPdmDN	0.8	1.09	1.48	0.55	0.58	305
dvDNdmUP	0.77	1.05	1.42	0.3	0.77	305
dvDNdmDN	0.94	1.2	1.54	1.45	0.15	305

**Multivariate (adj.for size+age+stage+ER-status)**						
**progZ**	**LI**	**HR**	**HI**	**z**	**P**	**n**
dvUPdmUP	0.97	1.28	1.68	1.80	0.07	296
dvUPdmDN	0.80	1.15	1.66	0.77	0.44	296
dvDNdmUP	0.80	1.09	1.50	0.58	0.56	296
dvDNdmDN	0.82	1.07	1.39	0.47	0.64	296

Supplementary Table 6: Univariate and multivariate Cox-regression analysis. Cox-regression results of the progression Z-scores for the four different classes of DVMCs, as indicated, with overall survival, in both univariate and multivariate analysis. Columns label lower 95% confidence interval (LI) for the Hazard Ratio, the Hazard Ratio itself (HR), its higher 95% CI (HI), z-statistic (z), chi-square score test P-value (P) and the number of samples (n).

Univariate	LI	HR	HI	z	P	n
**Frac(FD)**	1.03	1.44	2.00	2.13	0.03	305

Multivariate (adj.for size+age+stage+ER-status)	LI	HR	HI	z	P	n
**Frac(FD)**	0.90	1.30	1.87	1.40	0.16	296

Supplementary Table 7: Survival Cox-regression analysis for the fraction of altered field defects (Frac(FD)). Cox-regression results of a score, calculated as the fraction of hypervariable DVMCs which exhibit significant DNAm deviations in a cancer sample (Frac(FD)), as indicated, with overall survival, in both univariate and multivariate analysis. Columns label lower 95% confidence interval (LI) for the Hazard Ratio, the Hazard Ratio itself (HR), its higher 95% CI (HI), z-statistic (z), chi-square score test P-value (P) and the number of samples (n).

Univariate progZ	LI	HR	HI	z	P	n
dvUPdmUP	1.06	1.37	1.78	2.38	0.016	154
dvUPdmDN	0.76	0.99	1.3	-0.05	0.96	154
dvDNdmUP	0.74	0.98	1.29	-0.17	0.86	154
dvDNdmDN	0.96	1.21	1.53	1.64	0.1	154
**FracFD**	0.96	1.32	1.80	1.73	0.08	154

Supplementary Table 8: Univariate Cox-regression analysis in the untreated TCGA cohort. Cox-regression results of the progression Z-scores for the four different classes of DVMCs, as indicated, as well as that of the Frac(FD) score, with overall survival, in the untreated (i.e. no chemo or anti-hormone therapy) subset of the breast cancer TCGA. Columns label lower 95% confidence interval (LI) for the Hazard Ratio, the Hazard Ratio itself (HR), its higher 95% CI (HI), z-statistic (z), chi-square score test P-value (P) and the number of samples (n).

	progDev (C-NADJ) dvUPdmUP	progDev (C-NADJ) dvUPdmDN	progDev (C-NADJ) dvDNdmUP	progDev (C-NADJ) dvDNdmDN
**ER**	0.968	0.307	0.762	0.805
**PR**	0.42	0.699	0.046	0.084
**HER2**	0.003	0.369	0.841	0.389
**KI67**	0.172	0.616	0.237	0.689
**Death/Alive**	0.948	0.19	0.981	0.784
**Tumor Size**	0.381	0.155	0.205	0.829
**Patient Age**	0.362	0.127	0.424	0.338

Supplementary Table 9: Association of individualized progression deviation scores of DVMCs with clinical phenotypes P-values of association between clinical characteristics of the breast cancers with matched normals (n=42) with the individualized progression deviation scores (progDev) for the four different classes of DVMCs, as indicated

Supplementary References

1. Teschendorff AE, Zhuang J, Widschwendter M: **Independent surrogate variable analysis to deconvolve confounding factors in large-scale microarray profiling studies.** *Bioinformatics* 2011, **27:**1496-1505.
2. Hair BY, Xu Z, Kirk EL, Harlid S, Sandhu R, Robinson WR, Wu MC, Olshan AF, Conway K, Taylor JA, Troester MA: **Body mass index associated with genome-wide methylation in breast tissue.** *Breast Cancer Res Treat* 2015, **151:**453-463.
3. Johnson KC, Koestler DC, Fleischer T, Chen P, Jenson EG, Marotti JD, Onega T, Kristensen VN, Christensen BC: **DNA methylation in ductal carcinoma in situ related with future development of invasive breast cancer.** *Clin Epigenetics* 2015, **7:**75.
4. Abba MC, Gong T, Lu Y, Lee J, Zhong Y, Lacunza E, Butti M, Takata Y, Gaddis S, Shen J, et al: **A Molecular Portrait Of High-Grade Ductal Carcinoma In Situ (DCIS).** *Cancer Res* 2015.
5. Nazor KL, Altun G, Lynch C, Tran H, Harness JV, Slavin I, Garitaonandia I, Muller FJ, Wang YC, Boscolo FS, et al: **Recurrent variations in DNA methylation in human pluripotent stem cells and their differentiated derivatives.** *Cell Stem Cell* 2012, **10:**620-634.
